# Regulation of the Leucine Metabolism in *Mortierella alpina*

**DOI:** 10.3390/jof8020196

**Published:** 2022-02-18

**Authors:** Robin Sonnabend, Lucas Seiler, Markus Gressler

**Affiliations:** Pharmaceutical Microbiology, Friedrich-Schiller-University Jena, Leibniz Institute for Natural Product Research and Infection Biology, Hans-Knöll-Institute, Winzerlaer Strasse 2, 07745 Jena, Germany; robin.sonnabend@uni-jena.de (R.S.); lucas.seiler@stud.eah-jena.de (L.S.)

**Keywords:** branched-chain amino acid transaminase, α-isopropyl-malate synthase, leucine, basal fungus, *Mortierella*, feedback inhibition

## Abstract

The oleaginous fungus *Mortierella alpina* is a safe source of polyunsaturated fatty acids (PUFA) in industrial food and feed production. Besides PUFA production, pharmaceutically relevant surface-active and antimicrobial oligopeptides were isolated from this basal fungus. Both production of fatty acids and oligopeptides rely on the biosynthesis and high turnover of branched-chain-amino acids (BCAA), especially l-leucine. However, the regulation of BCAA biosynthesis in basal fungi is largely unknown. Here, we report on the regulation of the leucine, isoleucine, and valine metabolism in *M. alpina*. In contrast to higher fungi, the biosynthetic genes for BCAA are hardly transcriptionally regulated, as shown by qRT-PCR analysis, which suggests a constant production of BCAAs. However, the enzymes of the leucine metabolism are tightly metabolically regulated. Three enzymes of the leucine metabolism were heterologously produced in *Escherichia coli*, one of which is inhibited by allosteric feedback loops: The key regulator is the α-isopropylmalate synthase LeuA1, which is strongly disabled by l-leucine, α-ketoisocaproate, and propionyl-CoA, the precursor of the odd-chain fatty acid catabolism. Its gene is not related to homologs from higher fungi, but it has been inherited from a phototrophic ancestor by horizontal gene transfer.

## 1. Introduction

**Figure 1:**
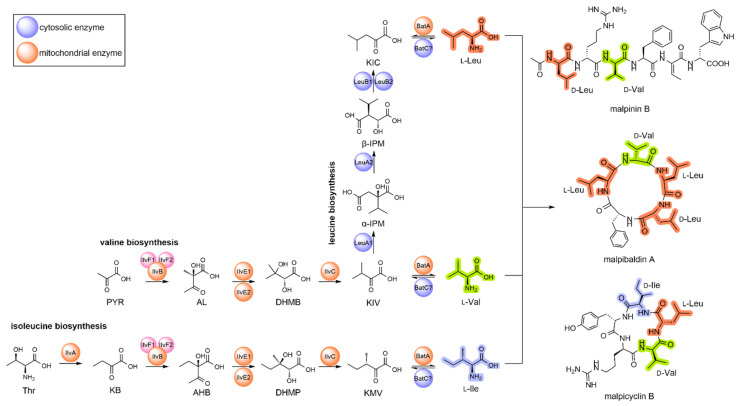
Proposed BCAA biosynthesis pathway and interconnection to the secondary metabolism in *M. alpina*. The proteins that participate in the pathway are (in alphabetical order): BatA (mitochondrial BCAA aminotransferase), BatC (cytosolic BCAA aminotransferase), IlvA (threonine dehydratase/deaminase), IlvB (acetolactate synthase, large subunit), IlvC (dihydroxy acid dehydratase), IlvE (acetohydroxy acid reductoisomerase), IlvF (acetolactate synthase, small subunit), LeuA1 (α-isopropylmalate synthase), LeuA2 (isopropyl malate isomerase), and LeuB1/2 (two β-isopropyl malate dehydrogenases). The precursors, intermediates, and final products are (in alphabetical order): AHB ((*2S*)-2-aceto-2-hydroxy-butyrate), AL ((*2S*)-acetolactate), DHMB ((*2R)*-2,3-dihydroxy-3-methyl-butyrate), DHMP ((*2R, 3R)-*2,3-dihydroxy-3-methyl-pentanoate), α-IPM ((*2S*)-α-isopropyl malate), Ile (l-isoleucine), β-IPM ((*2R, 3S*)-β-isopropyl malate), KB (α-ketobutyrate), KIC (α-ketoisocaproate), KIV (α-keto-isovalerate), KMV ((*3S*)-α-keto-3-methyl-valerate), Leu (l-leucine), PYR (pyruvate), Thr (l-threonine), and Val (l-valine). Mitochondrial and cytosolic enzymes are highlighted in ochre and azure, respectively. For more details on the proteins, refer to Table 1.

The biosynthesis of branched-chain amino acids (BCAAs), i.e., l-leucine (Leu), l-isoleucine (Ile), and l-valine (Val), is one of the most complexly regulated systems in biochemistry. It has been well-studied in the ascomycete *Saccharomyces cerevisiae* [1], and anecdotal descriptions exist for filamentous fungi, such as *Aspergillus fumigatus* [2] and *Pyricularia* (syn. *Magnaporthe* *oryzae*) [3], but it is much less understood for basal fungi such as *Mortierella alpina* [4,5]. The anabolic route to BCAAs in *S. cerevisiae* requires at least 17 cytosolic and mitochondrial enzymes and transporters (Figure 1 and Table 1). The biosynthesis of Ile is initiated by deamination of threonine by the desaminase Ilv1. The subsequent three steps, i.e., the condensation with pyruvate, the reduction and the dehydration by Ilv2/Ilv6, Ilv5, and Ilv3, respectively, results in α-ketomethylvalerate (KMV) [1], which, in turn, is transaminated to Ile by two BCAA aminotransferases (Bat1 or Bat2) [6]. In a similar reaction cascade, pyruvate acts as the precursor of Val biosynthesis using the same enzyme set [1], in which α-ketoisovalerate (KIV) is the final substrate for Bat1 to give Val. Interestingly, KIV is an important biosynthetic branch for Leu biosynthesis as well, as it is also a substrate for two acetyl-CoA-dependent α-isopropylmalate synthases (IPMS), Leu4 and Leu9, which provide α-isopropylmalate as the precursor for Leu biosynthesis [7]. Further isomerization and reduction by Leu1 and Leu2 result in α-ketoisocaproate (KIC), which is finally transaminated by Bat1 or Bat2 to Leu. BCAA production is primarily transcriptionally regulated, especially by the α-isopropylmalate-dependent transcription factor Leu3 [8]. In addition, some enzymes, e.g., the IPMS, are also metabolically regulated by negative feedback loops [7,9]. The knowledge on BCAA metabolism for model organisms, such as yeast and *Aspergilli*, contrasts the paucity of data for early diverging fungi. Even though they are industrially relevant producers of lipids or proteins, their BCAA enzymes have never been biochemically characterized in vitro.

The oleaginous basal fungus *M. alpina* is an important resource for natural polyunsaturated fatty acids (PUFA), including arachidonic acid, dihomo-γ-linolenic acid, and γ-linoleic acid [10]. *Mortierella* PUFAs are industrially used as nutritional supplements in human food or in animal feed [11]. The PUFA production can be boosted by supplementation with animal fat by-products [12] or odd-chain alkanes that increase the yield of C15, C17, and C19 odd-chain fatty acids [13]. Moreover, nitrogen deprivation can drive PUFA production by redirection of the glycolytic flux of carbon backbones into fatty acid biosynthesis because decomposition of some amino acids, including BCAAs, is accompanied with the release of acetyl-CoA, the building block for fatty acids [14]. Indeed, the BCAA pathway enzymes are coregulated with the lipid metabolism in oleaginous fungi [5]. Homologous overproduction of the Leu2-homolog LeuB1 in *M. alpina* (Figure 1) enhanced the supply of acyl-coenzymes A, such as acetyl-CoA (Ac-CoA), propionyl-CoA (Prop-CoA), and malonyl-CoA (Mal-CoA) and, in turn, elevated the PUFA product yields up to 30% [5]. Hence, knowledge about the BCAA regulation is required for an optimized PUFA production.

**Table 1:** Proteins of Leu metabolism in *M. alpina*. Proteins with verified function (*Saccharomyces cerevisiae*, *Pyricularia oryzae*, *Aspergillus fumigatus*, *Phycomyces blakesleeanus*) are shown. *, predicted function. +, BlastP analysis according to Bat3 from *P. oryzae* [3].

	Protein Function	Homolog in *S. cerevisiae* [1]	Homolog in *P. oryzae* [3]	Homolog in *A. fumigatus* [2,15]	Homolog in *P. blakesleeanus* [4]	Predicted Protein in *M. alpina*	e Value to *S. cerevisiae* Homologs	Encoding Contig	Signal Peptide Target-P [16]	Signal Pept. Mitofate [17]
**early steps in** **Leu biosynthesis**	threonine deaminase	ILV1				**IlvA**	1.05 × 10^−158^	ADAG01001032	yes	no
	acetohydroxy acid synthase catalytic subunit	ILV2	ILV2 *			**IlvB**	0	ADAG01000936	IRF-RS	yes
	acetohydroxy acid synthase regulatory subunit	ILV6	ILV6 *			**IlvF1** **IlvF2**	8.91 × 10^−73^ 8.91 × 10^−69^	ADAG01000936 ADAG01001016	no yes	no yes
	bifunctional acetohydroxy acid reductoisomerase	ILV5	ILV5 *			**IlvE1** **IlvE2**	2.32 × 10^−159^ 5.25 × 10^−157^	ADAG01000936 ADAG01001016	TRG-VK TRG-VK	yes yes
	dihydroxy acid dehydratase	ILV3	ILV3 *			**IlvC**	1.56 × 10^−153^	ADAG01001099	HRY-ST	no
**late steps in** **Leu biosynthesis**	α-isopropylmalate synthase I	LEU4S/L	LEU4	LeuC	LeuA	**LeuA1**	8.14 × 10^−23^	ADAG01001098	no	no
	α-isopropylmalate synthase II	LEU9	LEU9							
	α-isopropylmalate isomerase/dehydratase	LEU1	LEU1	LeuA		**LeuA2**	0	ADAG01001081	no	no
	β-isopropylmalate dehydrogenase	LEU2	Leu2A	Leu2A		**LeuB1** **LeuB2**	1.38 × 10^−112^ 1.83 × 10^−36^	ADAG01001016 ADAG01000512	no no	no no
	Zn_2_Cys_6_ transcription factor	LEU3	LEU3	LeuB		**LeuC1** **LeuC2**	5.57 × 10^−2^ 5.78 × 10^−2^	ADAG01000955 ADAG01000881	no no	no no
	CoA importer	LEU5				**LeuE1** **LeuE2**	2.33 × 10^−60^ 1.45 × 10^−47^	ADAG01001074 ADAG01001044	no no	no no
	Iron-sulfur cluster transporter	ATM1				**AtmA1** **AtmA2**	2.19 × 10^−154^ 1.50 × 10^−92^	ADAG01000995 ADAG01001098	no no	no no
	BCAA aminotransferase	BAT1 BAT2 -	BAT1 BAT2 BAT3 *			**BatA** **-** **BatC**	1.69 × 10^−52^ - 1.61 × 10^−32^	ADAG01001070 - ADAG01000958	RFY-YR - no	yes - no

Beside PUFA production, recent investigations highlight a previously underestimated secondary metabolism (SM) in *M. alpina*. The fungus produces numerous linear or cyclic oligopeptides including the biosurfactant malpinins [18,19], the antimycobacterial agent calpinactam [20], and the antibiotic cyclopentapeptides malpicyclins and malpibaldins [21]. The oligopeptide biosyntheses were assigned to non-ribosomal peptide synthetases, whose genes have been, most likely, transferred from bacterial endosymbionts by horizontal gene transfer (HGT) [21,22,23]. Indeed, both enzymes show typical bacterial-like dual epimerization/condensation (E/C) domains that facilitate the incorporation of d-configured amino acids, including BCAAs (Figure 1). Malpinin A, i.e., the major metabolite in *M. alpina*, contains two Leu moieties per molecule and is produced in titers up to 5% of the total fungal dry weight [19]. Hence, a high supply of BCAAs is required for efficient production of secondary metabolites in *M. alpina*.

The regulation of the BCAA metabolism has been intensely studied for higher fungi. In contrast, little is known for basal fungi, which are industrially important producers of fatty acids or secondary metabolites. In this study, we investigate the transcriptional and biochemical regulation of the BCAA metabolism and its connection to the malpinin biosynthesis in *M. alpina*. Contrasting higher fungi, basal fungi hardly transcriptionally regulate their BCAA biosynthetic genes ds. Hence, we aimed at the biochemical characterization of the main enzymes to unroll their metabolic regulon. The constitutively produced BCAA aminotransferase is hardly regulated because it is required for both BCAA anabolism and catabolism. In contrast, a complex transcriptional and metabolic control was observed for the α-isopropyl malate synthase in *M. alpina*. This key enzyme of Leu biosynthesis is tightly regulated via inhibition by its end-product l-leucine, products of the lipid catabolism, and BCAA degradation.

## 2. Materials and Methods

### 2.1. Microbiological Methods

*M. alpina* ATCC32222 was maintained on MEP (15 g L^−1^ malt extract, 3 g L^−1^ peptone, 18 g L^−1^ agar) plates. To collect biomass for inoculation of liquid cultures, *M. alpina* was cultivated for 7 days, on MEP agar plates, at 25 °C and an additional 14 days at 7 °C. Mycelium was collected by addition of 20 mL sterile water to each plate and 1.2 mL of the mycelial suspension was used for inoculation of 100 mL *Aspergillus* minimal medium (AMM) supplemented with 100 mM d-glucose, 20 mM d-glutamine, and 10 mM l-leucine, l-isoleucine, and/or l-valine, if required [24]. Liquid cultures were maintained at 180 rpm for 3, 5, or 7 days. *Escherichia coli* XL-1 blue and BL21 (DE3) were used for plasmid propagation and protein production, respectively, and they were maintained in LB medium (10 g L^−1^ peptone, 10 g L^−1^ NaCl, 5 g L^−1^ yeast extract) with 50 µg mL^−1^ of kanamycin, if required.

### 2.2. gDNA and RNA Isolation, cDNA Synthesis and Expression Analysis

Fungal mycelium was lysed with glass beads by a FastPrep Homogenizer (MP Bio) for 2 min at 4.5 m s^−1^. Genomic DNA was isolated as described [21]. RNA was isolated with the SV Total RNA Isolation System (Promega) using the manufacturer′s protocol. RNA (1 µg) was treated with Baseline-ZERO DNase (Lucigen) and was reversely transcribed to cDNA by the RevertAid RT kit (Thermo) using anchored oligo-(dT)_20_ primers. Expression analysis was carried out with an AnalytikJena qTower^3^, using the qPCR Mix EvaGreen (Bio&SELL) and oligonucleotides with a minimum primer efficiency of 92 % (Table S1). Amplification procedure: initial denaturation, 95 °C, 15 min; 40 cycles of amplification (95 °C, 15 s; 60 °C, 20 s; 72 °C, 20 s). A melting curve was monitored by heating from 60 to 95 °C. The housekeeping reference genes, encoding α-actin (*actB*) and glycerinaldehyde-3-phosphate dehydrogenase (*gpdA*), served as internal standard. Gene expression levels were determined as described [25].

### 2.3. Heterologous Protein Production and Size Exclusion Chromatography

The construction of expression plasmids, encoding BatA (with and without mitochondrial import sequence), BatC and LeuA1, and the used oligonucleotides, are described in the Supplementary Materials and are listed in Tables S1 and S2. Production of the N-terminally His_6_-tagged proteins was performed in Escherichia coli BL21. Details on the production procedure, purification, determination of protein concentration, and size exclusion chromatography (SEC) are described in the Supplementary Materials.

### 2.4. Chromatographical Quantification of α-amino Acids, α-keto Acids and Malpinins

Details on the extraction procedure and chromatographical analyses are described in the Supplementary Materials. In brief, to determine the intracellular amino acids, 50–400 mg of lyophilized fungal biomass was extracted twice with an equal volume of methanol and extracts were analysed on a SeQuant ZIC-HILIC columns (Merck) using method A or method B (Table S3). BAT activity was determined based on a calibration curve with l-glutamate. Commercial l-amino acids and α-keto acids (Merck) served as references. For the quantification of malpinins, 50 mL of cell-free culture supernatant was extracted twice with equal volumes of ethylacetate, and after evaporation, residues were dissolved in 2 mL methanol. Analysis was performed on a Zorbax Eclipse Plus C18 RRHD (Agilent) column using HPLC method C (Table S3). A calibration curve of malpinin A (*m*/*z* 859 [M + H]^+^), ranging from 0 to 500 µg/mL, served as reference standard.

### 2.5. Determination of Enzyme Activities

BatA and BatC activities were determined in BCAA aminotransferase (BAT) buffer (50 µM pyridoxal phosphate (PLP), 50 mM Tris-HCl, pH 8.0). To determine kinetic parameters and alternative substrates, a discontinuous assay was performed: 190 µL master mix (5 mM α-ketoglutarate (KG), 130 nM BatA or BatC in BAT buffer) was mixed. In a two-step approach, the reaction was initially started by adding 10 µL of pools of α-amino acids (1 mM each). Subsequently, individual α-amino acids were tested (0–1 mM final concentrations). For the BCAA synthetic direction, KG was replaced by l-glutamate (Glu) and the reaction was started with 10 µL of α-keto acids KIC, KIV, and KMV (1 mM each). After 2, 5, 10, 15, 20, and 40 min at 25 °C, the reaction was stopped by shock-freezing in liquid nitrogen. The reaction mixture was lyophilized, dissolved in 100 µL methanol, centrifuged (10 min, 14,000× *g*), and the supernatant was subjected for quantification on a HILIC-UHPLC system, using a calibration curve with Glu as reference (Supplementary Materials). For the temperature optimum, the reaction mixture was incubated with Leu as substrate at 4–60 °C. The pH optimum was determined in BAT buffer between pH 7.0 and 9.5. To determine PLP dependence, PLP was omitted.

LeuA1 activity was determined in α-isopropylmalate synthase (IPMS) buffer (50 mM Tris-HCl, pH 8.0). To determine kinetic parameters and alternative substrates, a continuous assay in a 100 µL-scale was performed by detecting free thiol groups of coenzyme A (CoA) using Ellman’s reagent (5,5-dithio-bis-(2-nitrobenzoic acid), DTNB): 25 µL master mix (80 nM LeuA1 in IPMS buffer) were mixed. Next, 20 µL of alternative acyl CoA substrates (Ac-CoA, Prop-CoA, Mal-CoA, final concentrations 0–200 µM) and 5 µL α-keto acids (i.e., α-ketoisovalerate (KIV), α-ketobutyrate (KB), α-ketoglutarate (KG), or pyruvate at a final concentration of 0–200 µM) were added, and the reaction was started by the addition of 50 µL DTNB (final concentration 100 µM). Colorimetric detection of the released yellow product (2-nitro-5-thio-benzoic acid) was carried out in a 96-well plate at λ_abs_ = 412 nm and λ_abs_ = 800 nm as reference wavelength at 35 °C for 8 min in a Clariostar plate reader (BMG Labtech). The continuous assay was used to determine activity, kinetic parameters of substrates and inhibitors (*K*_M_, *k*_cat_, and *K*_I_), pH optimum, and metal ion dependencies towards divalent cations (MgSO_4_, MnCl_2_, CaCl_2_, CoCl_2_, or ZnAc_2_ at a final concentration of 0–5 mM) or monovalent cations (NaCl or KCl, 50–140 mM). To study the feedback inhibition of the enzyme, 0–5 mM BCAAs (Ile, Leu, Val) or KIC were added. The inhibition by CoA esters was tested in the presence of 0–200 µM Prop-CoA or Mal-CoA. To determine the temperature optimum, a discontinuous assay was performed: the reaction mixture (100 µL) was incubated at 4–60 °C, and reaction was stopped by the addition of 50 µL ethanol after 8 min incubation. After the addition of 50 µL DTNB (200 µM), the mixture was assayed colorimetrically, as described above.

## 3. Results

### 3.1. Identification of BCAA Biosynthetic Genes in M. alpina

**Figure 2:**
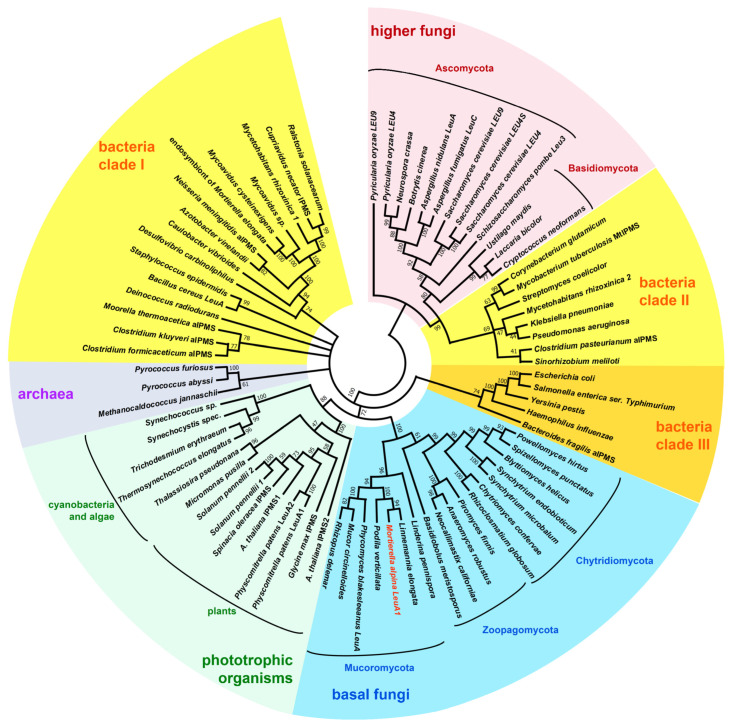
Phylogenetic analysis of IPMS enzymes from various species. The evolutionary history was inferred by using the Maximum Likelihood method and Le Gascuel 2008 model [29]. The percentage of trees in which the associated taxa clustered together is shown next to the branches. Initial tree(s) for the heuristic search were obtained automatically by applying Neighbor–Join and BioNJ algorithms to a matrix of pairwise distances, estimated using the JTT model, and then selecting the topology with superior log likelihood value. A discrete Gamma distribution was used to model evolutionary rate differences among sites (5 categories (+G, parameter = 2.0850)). The rate variation model allowed for some sites to be evolutionarily invariable ([+I], 2.39% sites). Evolutionary analyses were conducted in MEGA11 [30]. Note that basal fungi and higher fungi comprise a divergent phylogenetic origin. The *M. alpina* LeuA1 is highlighted in red. Enzymes that have been biochemically or genetically verified are indicated with their names. Other proteins are predicted. The accession numbers of the 77 analysed protein sequences are listed in Table S4.

To identify the biosynthetic genes involved in the Ile, Leu, and Val metabolism, a BLAST analysis was conducted for the reference genome of Mortierella alpina ATCC32222, using amino acid sequences of the well-studied enzymes from ascomycetes (*Saccharomyces cerevisiae* and *Pyricularia* (syn. *Magnaporthe*) *oryzae* as queries (Table 1, Figure 1). Interestingly, the genes of most mitochondrial enzymes, i.e., the early steps in Leu biosynthesis, converting pyruvate or threonine to the α-keto carboxylic acids KIV and KMV, respectively, are encoded in two partially duplicated gene clusters (*ilvB-ilvF1-ilvE1* and *ilvF2-ilvE2-leuB1*). Similarly, the genes for the cytosolic late steps in Leu biosynthesis are partially duplicated (*leuB1/2, leuE1/2*, *atm1/2*) but are not part of a gene cluster. In *S. cerevisiae* and *P. oryzae*, two paralogous genes encoding for a mitochondrial and cytosolic version of the α-isopropylmalate synthase (IPMS) can be found, denoted as *leu4* and *leu9*. However, only one *leu4* copy was identified in *M. alpina* (*leuA1*), but the overall similarity is comparatively low (e value: 8.14 × 10^−23^). This suggests that *leuA1* is not related to genes of higher fungi and might have been introduced by HGT from another genetic source. This has been suggested for the *leuA* gene from the basal fungus *Phycomyces blakesleeanus* [4]. Indeed, in an inter-kingdom phylogenetic analysis of LeuA proteins, the *M. alpina* LeuA1 clusters with homologs from other basal fungi, plants and cyanobacteria (Clade I), whilst LeuA proteins from Dikarya form a distantly separated cluster, sharing a bacterial sisterclade (Clade II) (Figure 2, Table S4). Hence, we concluded that IPMS´s from basal fungi, including Mucoromycota, Zoopagomycota, and Chytridiomycota, derived from a phototrophic ancestor. In contrast, IPMS´s from Dikarya may have originated from heterotrophic bacteria.

The final step in BCAA biosynthesis is the conversion of α-keto acids to Ile, Leu, and Val and is catalysed by branched-chain amino acid aminotransferases (BATs), whose genes are present in multiple copies in ascomycetes [26]. However, the *M. alpina* genome encodes solely one mitochondrial BAT-homologue (BatA). A paralogous gene encoding a cytosolic version—such as BAT2 in *S. cerevisiae* [6]—is missing. In yeast, the mitochondrial BAT1 is produced during BCAA biosynthesis, whilst the BAT2 gene is expressed during BCAA catabolism, resulting in an inverse regulation of both genes [27]. The presence of just one BAT homolog in *M. alpina* indicates a constant expression and, hence, constant abundance of BatA for both BCAA anabolism and catabolism. However, in the rice blast fungus *P. oryzae*, a third BAT is postulated (Bat3), [3] and a far related homologous *bat3* gene is encoded in the *M. alpina* genome as well (*batC*). Whilst the *ilv* and *leu* expression levels are regulated by a global, α-IPM-dependent Zn(II)_2_Cys_6_ transcription factor, Leu3/LeuB, in Dikarya [27,28], a homologous *leu3* gene, is not present in basal fungi, suggesting a different kind of regulation. This is further supported by the fact that well-conserved Leu3/LeuB binding sites (5′-CCGNNNNCGG-3′) [28] are not present in the *ilv* and *leu* promoter regions of *M. alpina*.

### 3.2. BCAA Uptake and Its Impact on Malpinin Biosynthesis

**Figure 3:**
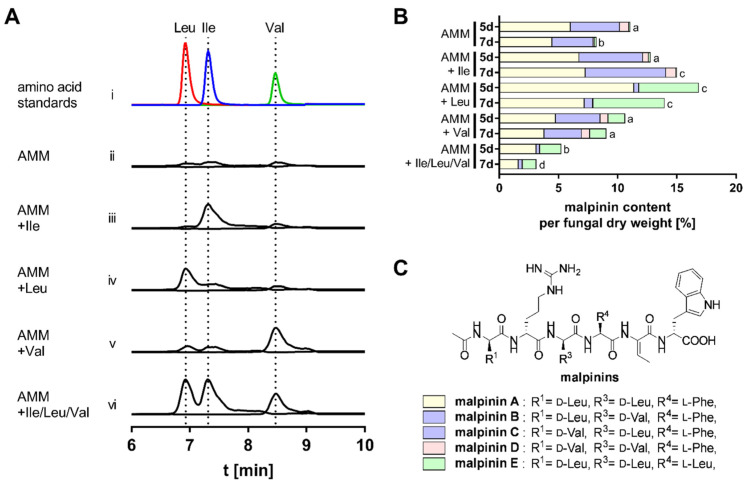
BCAA uptake in *M. alpina* and incorporation in malpinins. (**A**) UHPLC-MS profiles of cell extracts from lysed *M. alpina* after cultivation with additional BCAAs (Ile, Val, Leu). Commercial standards served as control (trace i). Uptake of BCAAs (traces ii-vi) is demonstrated by the extracted ion chromatograms for Val (r_t_ = 8.4 min, *m/z* 118.15 [M + H]^+^) and Leu/Ile (r_t_ = 6.9/7.2 min, *m/z* 132.18 [M + H]^+^) in an overlay mode. (**B**) Accumulated malpinin production in *M. alpina* by supplementation with or without Ile, Val, Leu, or all BCAAs in parallel. Cultures were harvested after 5 and 7 days, and malpinin content was determined from the supernatant by their AUCs in the EICs, using an authentic malpinin standard as reference. Values were normalized according to the fungal dry biomass and indicated as mg malpinins per g biomass. Data on total malpinin content were grouped in a compact letter display, in which samples sharing the same letter (a–d) are not significantly differently regulated to the reference condition (AMM-5d) (*p* < 0.05). Note that EIC signals of the isomeric compounds malpinin B and C (both *m/z* 845 [M + H]^+^) partially overlap, and, therefore, a combined quantification for both metabolites was carried out. (**C**) Chemical structures of malpinins A–E. For statistical analysis of quantification, see Table S5.

To decipher the regulation of BCAA biosynthesis pathways in *M. alpina*, the fungus was cultured in minimal medium in absence or presence of 10 mM Ile, Leu, Val, or all BCAAs. Uptake of the three BCAAs was monitored by UHPLC-MS (Figure 3A). Elevated levels of the respective BCAA was determined in presence of any of the amino acids, indicating that all BCAA are taken up by *M. alpina.* Interestingly, Leu supplementation resulted in an eight-fold and two-fold increased intracellular titer of Leu and Ile, respectively, suggesting an interconnection of Leu degradation and Ile biosynthesis. Inversely, when Val was supplemented, a two-fold elevated level of Leu is detectable, indicating a link between Val degradation and Leu anabolism. Hence, an excess of Val is rapidly recognized and readily converted to Leu.

**Figure 4:**
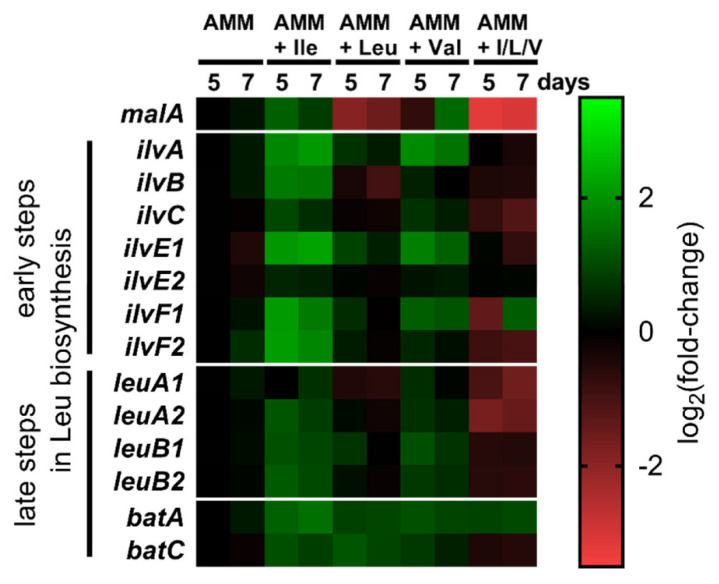
Heatmap of the expression profiles of the *M. alpina* BCAA and malpinin biosynthesis pathways based on qRT-PCR data. For details on BCAA genes and expression raw data, refer to Table 1, Tables S6 and S7. *malA*, malpinin synthetase gene. AMM, *Aspergillus* Minimal Medium, without or supplemented with 10 mM BCAAs; + I/L/V, + Ile + Leu + Val (10 mM each).

The impact of BCAAs on secondary metabolite production was monitored by the production rates of malpinins A–E, i.e., the most abundant non-ribosomal peptides in *M. alpina* (Figure 3B,C, Table S5). The qualitative composition of malpinins A–E can be altered by the variation of supplemented BCAAs [19]. Both Ile and Leu supplementation resulted in a slightly increased production rate of malpinins by up to 33%. The production of Leu-containing metabolites, malpinin A and E, was increased when Leu was fed to the cultures. Similarly, addition of Val shifted the metabolic profile towards Val-containing malpinin B, C, and D (Figure 3B), as described previously [19]. In agreement with the Val uptake experiments, suggesting a slight elevated Leu level (Figure 3A), a pronounced production of the *all*-Leu metabolite malpinin E was detectable. In sharp contrast, when the complete set of BCAAs was supplied, a severe reduction in malpinin biosynthesis was observed (51% after 5 days and 62% after 7 days of incubation, *p* < 0.001). This suggested, that malpinins are preferably produced under BCAA-limited conditions. Interestingly, according to qRT-PCR analysis, the expression of the corresponding malpinin synthase gene (*malA*) is strongly regulated by the presence of BCAAs (Figure 4). The presence of Ile and Val did not significantly affect malA expression, whilst Leu slightly repressed *malA* by factor 3.5 (*p* < 0.001), (Figure 4, Tables S6 and S7). Since Leu is the preferred substrate for MalA [22], this overcompensates the *malA* repression by Leu, and the titers of malpinins remained high. However, repression was enhanced when all competing BCAAs were supplemented (9.1-fold, *p* < 0.001) and malpinin content dropped (Figure 3B). In sum, among the BCAAs, Leu has the most striking impact on *malA* expression in *M. alpina*.

### 3.3. Transcriptional Regulation of BCAA Biosynthesis

To monitor the expression of the Leu biosynthesis genes, fungal cultures were supplemented with individual or all BCAAs and qRT-PCR expression analyses were performed (Figure 4, Tables S6 and S7). In general, the presence of BCAAs has only minor impact on the expression (maximal +/− 4-fold deregulation), suggesting a constant BCAA biosynthesis in M. alpina. The presence of Ile significantly upregulated (3–4 fold, *p* < 0.001) all genes required for Leu biosynthesis, indicating that Ile excess is equivalent to Leu limitation in the cell. Similarly, feeding of Val significantly (*p* < 0.01) induced expression of genes responsible for the early Leu biosynthetic steps (*ilvA, ilvE1*, and *ilvF1*). In contrast, presence of Leu did not significantly alter the expression levels of any of the biosynthetic genes, suggesting that breakdown of Leu requires at least in part the same set of enzymes as for the anabolic pathway. Moreover, repression by Leu is still evident and intensified, when all three BCAA were added. The genes for the early steps in Leu biosynthesis were unaffected but two genes for the late steps were downregulated, especially *leuA1* (3-fold, *p* < 0.001) and *leuA2* (3.5-fold, *p* < 0.001), suggesting that the encoded enzymes are the gatekeepers for the Leu biosynthesis. Therefore, Leu seems to be the major repressor for BCAA biosynthesis genes and overcomes the inducing effects by Ile or Val. Interestingly, the two potential BAT genes (*batA* and *batC*) are not significantly regulated by any of the supplementations, suggesting a requirement for both BCAA biosynthesis and degradation. This sharply contrasts the BCAA-dependent transcriptional regulation of BAT genes in other fungi [2,6,26].

In summary, the *M. alpina* genome encodes the complete subset of mitochondrial or cytosolic enzymes to produce BCAAs, but the genes are hardly transcriptionally regulated by Leu, contrasting observations in Dikarya. Hence, a more pronounced metabolic regulation was examined. Since basal fungi are hardly accessible for gene knock-out strategies, we aimed at the biochemical characterization of selected heterologously produced enzymes of the BCAA metabolism.

### 3.4. Biochemical Characterization of BatA

The BCAA aminotransferases (BAT) catalyse the final step in BCAA biosynthesis and the first step in BCAA degradation, i.e., the transamination of an α-keto acid to its respective BCAA and vice versa. Interestingly, genomes of ascomycetes usually encode at least two—usually inversely regulated—genes for BATs for anabolic BCAA biosynthesis and BCAA catabolism, respectively [26]. However, the genome of *M. alpina* encodes only one enzyme (BatA, 45.2 kDa). In *P. oryzae*, a third distantly related BAT is postulated (Bat3), and a similar enzyme (BatC, 39.9 kDa) is encoded in the *M. alpina* genome. According to Target-P [16] and MitoFate [17], BatA is probably a mitochondrial enzyme containing a 34 aa long, N-terminal mitochondrial import sequence (MIS), whilst BatC is a cytosolic protein. An inter-kingdom phylogenetic analysis revealed that BatA is part of a cluster among other fungal BATs (Figure S1, Table S8). In contrast, BatC grouped together with plant counterparts, and its gene is most likely plant-derived by HGT, as suggested for the other fungal leucine biosynthetic genes [4,31].

Two versions of BatA, with or without MIS (BatA-L and BatA-S), and BatC were heterologously produced and purified from *E. coli* (Figure S2). During purification it turned out that BatA-S was more stable than BatA-L. According to size exclusion chromatography (SEC), BatA-S is a monomeric enzyme in solution with a total size of 374 aa (41.6 kDa, Figure S3), which is similar to other fungal BATs [26].

**Figure 5:**
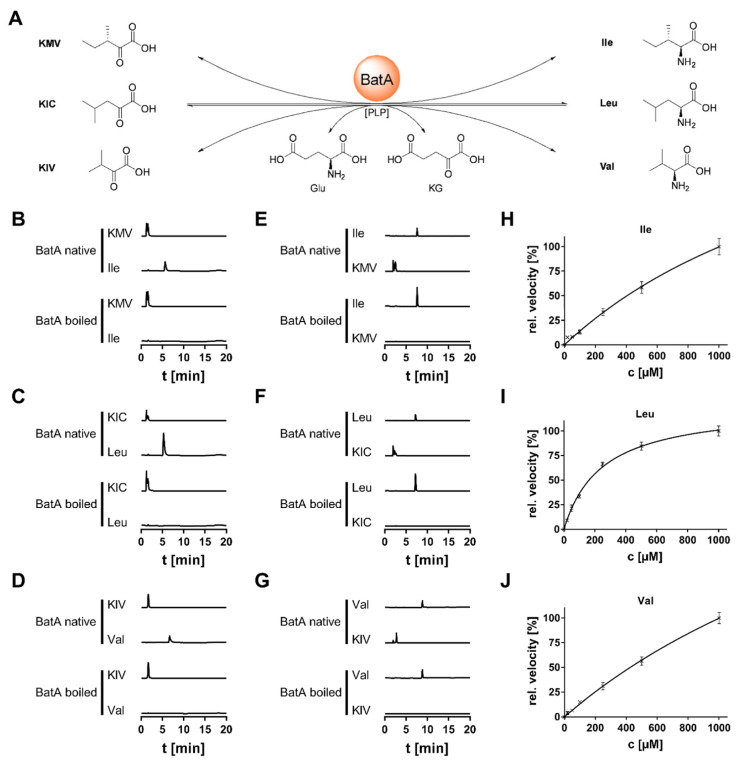
Conversion of α-keto acids and BCAAs by BatA from *M. alpina*. (**A**) Schematic representation of the BatA-catalysed reaction. (**B**–**G**) Extracted ion chromatograms of BatA reaction mixtures using α-keto acids (**B**–**D**) or their corresponding BCAAs (**E**–**G**) as substrates. The reaction was carried out with both the native and heat-inactivated enzyme. Commercial standards served as references. Note that branched-chain keto acids undergo stabilized keto-enol tautomerisation under physiologic conditions, resulting in a twin peak. (**H**–**J**) Michaelis–Menten kinetics for Ile (**H**), Leu (**I**), and Val (**J**). For kinetic parameters refer to Table 2.

A novel HILIC-UHPLC-MS-based enzyme assay was developed to detect all substrates and products at once in the BAT reaction mixture (Figure 5, Table S3). BatA-L did not show any turnover when BCAAs were applied as substrates. In contrast, when its N-terminal signal sequence was omitted (BatA-S), an active enzyme was produced (Figure S3). BatA required buffer systems with alkaline pH values (pH 8.0), which is a typicial feature for enzymes of the mitochondrial matrix [32] (Figure S4). Its temperature optimum of 25 °C is characteristic for enzymes from cryophilic fungi such as *Mortierellaceae* [33]. The enzyme converted all BCAAs (l-Ile, l-Leu, l-Val) and their respective α-keto acids in a PLP-dependent manner (Figure 5A–G and Figure S5). PLP reversibly binds to a highly conserved Lys residue in the substrate binding pocket of BATs [34], which is also present in BatA (Lys^345^). Some side activity could be detected with structurally similar amino acids such as l-Met and l-Phe (data not shown). In contrast, BatC was inactive, and neither converted any BCAA (Figure S5) nor any other proteinogenic l-amino acid (data not shown). These data, in combination with its low expression (Figure 4), suggested that *batC* is a pseudo-gene.

For reasons of comparability with literature data, kinetic values for BatA was determined in catabolic direction (Table 2 and Figure 5H–J). According to their *K*_M_ values, substrate affinity for Leu is twelve-fold higher than for Val. This is similar to data about mitochondrial BAT enzymes from animals and plants [35,36,37]. However, BatA also discriminates between Leu and Ile by one order of magnitude, which results in a three-fold lower *k*_cat_/*K*_M_ value for Leu. Hence, Leu biosynthesis and degradation is the favoured reaction of BatA. Val/Ile biosynthesis is solely localized in the mitochondrium, and its α-keto acids, KIV and KMV, are produced in the intimate presence of BatA. Conversely, the production of the Leu precursor KIC is localizied in the cytoplasm, and Leu production by BatA depends on the KIC production rates in the cytoplasm and its mitochondrial import.

**Table 2:** Kinetic parameters of BatA from *M. alpina* and related enzymes.

Organism	Enzyme	Substrate	*K*_M_ [µM]	*k*_cat_ [s^−1^]	*k*_cat_/*K*_M_ [µM^−1^ s^−1^]	Ref.
*Mortierella alpina*	BatA	Ile	2091.0 ± 505.1	49.67 ± 8.79	0.024	
		Leu	245.7 ± 18.6	17.73 ± 0.50	0.072	
		Val	2830.0 ± 435.5	84.87 ± 10.22	0.029	
*Rattus norvegicus*	BCATm	Ile	1300	n.d.	n.d.	[35]
		Leu	1600	n.d.	n.d.	
		Val	7700	n.d.	n.d.	
*human*	BCATm	Ile	600 ± 20	80 ± 1	0.165	[37]
		Leu	1210 ± 120	115 ± 11	0.088	
		Val	6100 ± 110	68 ± 2	0.011	
	BCATc	Ile	770 ± 20	172 ± 9	0.223	
		Leu	600 ± 40	132 ± 7	0.220	
		Val	2400 ± 90	122 ± 8	0.051	
*Arabidopsis thaliana*	BCAT3	Ile	140 ± 30	n.d.	n.d.	[36]
		Leu	140 ± 40	n.d.	n.d.	
		Val	1380 ± 100	n.d.	n.d.	
*Solanum lycopersicum*	BCAT-1	Ile	670 ± 90	40.8	0.061	[38]
	mitochondrial	Leu	560 ± 40	39.1	0.070	
		Val	1000 ± 100	50.5	0.050	
	BCAT-5	Ile	3200 ± 200	163.0	0.051	
	cytosolic	Leu	1800 ± 100	118.0	0.066	
		Val	2600 ± 200	123.0	0.047	

### 3.5. Biochemical Characterization of LeuA1

**Figure 6:**
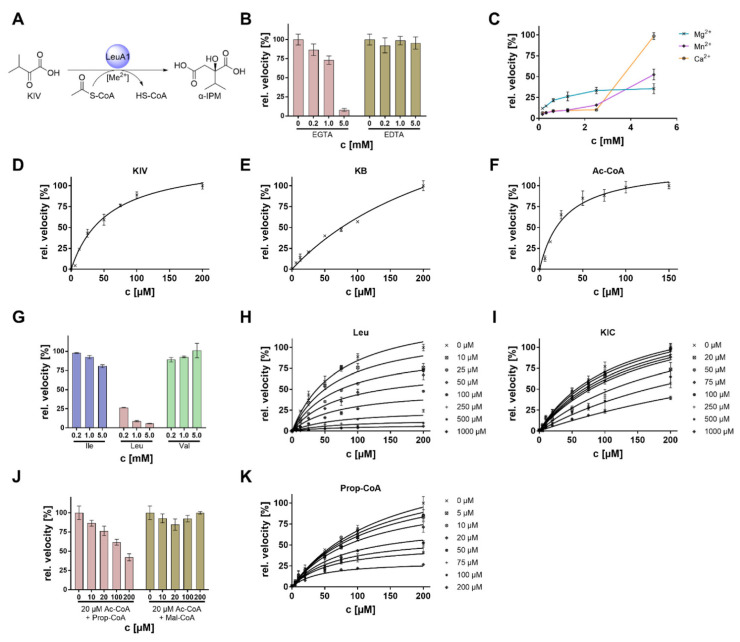
Metabolic regulation of the α-isopropyl malate synthase LeuA1. (**A**) Schematic representation of the LeuA1-catalyzed reaction. (**B**) Inhibition of LeuA1 by addition of chelators EGTA and EDTA. (**C**) Restoration of LeuA1 activity by additional divalent ions in the presence of 5 mM EGTA. Note that Mg^2+^ restores LeuA1 at low titers, whilst Mn^2+^ and Ca^2+^ require higher concentrations. (**D**–**F**) Michaelis-Menten kinetics for the substrates KIV (**D**), KB (**E**), and acetyl-CoA (**F**). (**G**) Inhibition of LeuA1 by addition of BCAAs. Note that LeuA1 is selectively inhibited by Leu. (**H**,**I**) Dose-dependent inhibition of LeuA1 by addition of Leu (**H**) and its precursor KIC (**I**). (**J**) Inhibition of LeuA1 activity by addition of propionyl-CoA and malonyl-CoA. Reactions were carried out in the presence of 20 µM acetyl-CoA as co-substrate. LeuA1 is selectively inhibited by propionyl-CoA. (**K**) Dose-dependent inhibition of LeuA1 by addition of propionyl-CoA. For kinetic parameters, refer to Table 3 and Table 4.

The α-isopropylmalate synthase (IPMS) LeuA1 is a cytosolic acetyl-CoA dependent C_2_-transferase that transfers an acetyl moiety to KIV to produce the Leu precursor α-IPM (Figure 6A and Table 1). IPMS acts as a gatekeeper enzyme in Leu anabolism on the one hand and is the branch to the Val biosynthesis on the other. It is strictly regulated across all biological kingdoms including bacteria [39], higher fungi [15], and plants [40]. Phylogenetically, IPMS´s fall into two divergent groups [4]. IPMSs from basal fungi are not related to counterparts from higher fungi (Dikarya), and they may be evolutionarily descended from photosynthetic organisms (Figure 2). Hence, *M. alpina* LeuA1 was biochemically characterized as a plant-derived IPMS from fungi, with a particular focus on its regulatory features.

The enzyme was heterologously produced in *E. coli* and purfied as N-terminally His_6_-tagged protein (Figure S2). IPMSs form homo- or heterodimers in solutions, as shown for enzymes of *M. tuberculosis* and *S. cerevisiae* [7]. SEC analysis indicated a hexameric structure of LeuA1 with a total molecular weight of 375 kDa (monomer 62.5 kDa, Figure S3). LeuA1 activity is optimal at pH 8.0 and shows a broad temperature stability up to 60 °C with an optimum at 35 °C (Figure S6). Several α-keto acids have been tested, and KIV and α-ketobutyrate (KB) are similarly converted when incubated with acetyl-CoA (Table 3 and Figure 6A,D,E). Both substrates show extraordinarily high turnover rates that exceeds values from known IPMS by a factor 8 to 12. Whilst the *Thermus thermophilus* IPMS converts KIV, and pyruvate to a similar extent (Table 3) [9], LeuA1 does not: neither pyruvate nor its stucturally related compound α-ketoglutarate (KG) are substrates for LeuA1. Among alternative acyl donors, the enzyme clearly accepts acetyl-CoA over malonyl- and propionyl-CoA (Figure S7).

**Table 3:** Kinetic parameters of LeuA1 from *M. alpina* and related enzymes. Ac-CoA, acetyl-CoA; KB, α-ketobutyrate; KG, α-ketoglutarate; KIV, α-ketoisovalerate; Mal-CoA, malonyl-CoA; Prop-CoA, propionyl-CoA; Pyr, pyruvate.

Organism	Enzyme	Substrate	*K*_M_ [µM]	*k*_cat_ [s^−1^]	*k*_cat_/*K*_M_ [µM^−1^ s^−1^]	Ref.
*Mortierella alpina*	LeuA1	KIV	56.9 ± 6.3	26.57 ± 1.20	0.467	
		KB	280.8 ± 45.0	47.35 ± 5.41	0.169	
		KG	Not a substrate	n.d.	n.d.	
		Pyr	Not a substrate	n.d.	n.d.	
		Ac-CoA	28.6 ± 4.1	24.98 ± 1.16	0.873	
		Prop-CoA	Not a substrate	n.d.	n.d.	
		Mal-CoA	Not a substrate	n.d.	n.d.	
*Mycobacterium tuberculosis*	MtIPMS	KIV	12 ± 1	3.50 ± 0.10	0.292	[39]
		KB	410 ± 180	7.00 ± 1.30	0.012	
		KG	n.d.	n.d.	n.d.	
		Pyr	9500 ± 1680	6.10 ± 0.50	0.001	
		Ac-CoA	136 ± 5	2.10 ± 0.10	0.015	
		Prop-CoA	220 ± 40	0.04 ± 0.003	0.0002	
		Mal-CoA	n.d.	n.d.	n.d.	
*Mycobacterium tuberculosis*	IPMS-2CR	KIV	261	1.17	0.005	[41]
		Ac-CoA	568	2.22	0.004	
	IPMS-14CR	KIV	35	0.52	0.015	
		Ac-CoA	27	0.61	0.023	
*Thermus thermophilus*	TTC0849	KIV	54.0 ± 5.8	1.20 ± 0.03	0.022	[9]
		Pyr	130.0 ± 12.0	1.20 ± 0.03	0.009	
		Ac-CoA	23.0 ± 2.5	1.70 ± 0.07	0.073	
*Arabidopsis thaliana*	IPMS1	KIV	304.0 ± 68.0	2.4 ± 0.21	0.008	[40]
		Ac-CoA	45.0 ± 10.0	2.2 ± 0.21	0.047	
	IPMS2	KIV	279.0 ± 68.0	2.3 ± 0.17	0.008	
		Ac-CoA	16.0 ± 10.0	1.9 ± 0.11	0.120	

**Table 4:** Kinetic parameters of LeuA1 inhibitors. Reactions were carried out in the presence of the substrates KIV and Ac-CoA. # α value was determined for mixed type inhibitor Prop-CoA (α < 1 uncompetitive, α = 1 non-competitive, α > 1 competitive).

Organism	Enzyme	Inhibitor	*K*_M_ [µM]	*K*_I_ [µM]	α ^#^	Comment
*Mortierella alpina*	LeuA1	Ile	n.d.	n.d.	n.d.	slight inhibition
		Leu	72.6 ± 4.8	53.0 ± 2.5	n.d.	non-competitive inhibition
		Val	n.d.	n.d.	n.d.	no inhibition
		KIC	124.5 ± 13.1	293.3 ± 30.4	n.d.	competitive inhibition
		Prop-CoA	145.2 ± 11.0	320.36 ± 121.17	0.14	mixed type inhibition

### 3.6. Complex Metabolic Regulation of the Key Enzyme LeuA1

*Regulation by metall ions.* Many IPMS enzymes are activated by monovalent cations such as potassium ions [42]. However, LeuA1 is neither activated nor inhibited by elevated concentrations of sodium or potassium ions (Figure S8). In contrast, when the chelating agent EGTA was added to the reaction mixture, activity dropped by 90%. This was not observed when EDTA was used instead (Figure 6B). Both EDTA and EGTA chelate preferrably Mg^2+^, but EGTA additionally traps other divalent ions such as Ca^2+^ [43], indicating that the Mg^2+^ can be replaced by other ions. Indeed, the reduced activity by adding EGTA can be restored by the addition of various divalent metal ions such as Mg^2+^, Ca^2+^ and Mn^2+ (^Figure 6C), whilst Zn^2+^ and Co^2+^ are strong inhibitors in the micromolar range (Figure S9). LeuA1 shares residues (Asp^18^, His^217^, and His^219^) known to coordinate divalent cations [42]. Hence, LeuA1 is dependent on exchangeable divalent cations.

**Figure 7:**
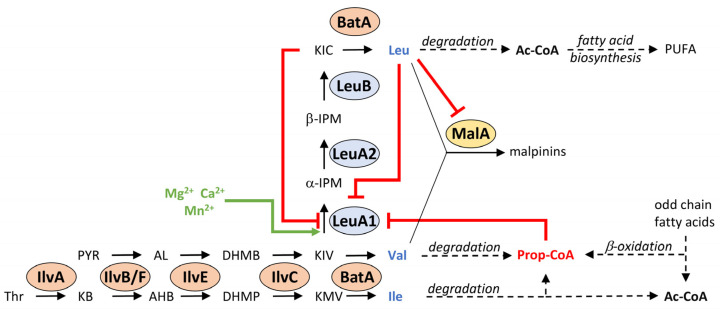
Schematic representation of the regulation of the BCAA and malpinin biosynthetic pathways in *M. alpina.* Enzymes and metabolic intermediates are listed in Figure 1. Ac-CoA, acetyl-coenzyme A; MalA, malpinin synthetase; Prop-CoA, propionyl-coenzyme A; PUFA, even-chain polyunsaturated fatty acids. Arrows and lines: continuous line, one catalytic step; dashed line, multiple catalytic steps; green bold lines, induction by metal ions; red bold lines, inhibition by intermediates or products.

*Feedback inhibition by Leu and KIC*. LeuA1 converts KIV and KB in an acetyl-CoA depended manner (Figure 6D–F). However, IMPS are well known to be feedback-regulated by their anabolic final product Leu [44]. Indeed, whilst Val and Ile hardly affect LeuA1, the presence of Leu dramatically impaired its activity (Figure 6G and Figure S10). The presence of Leu only slightly raised the *K*_M_ (Figure 6H and Figure S10), but *k*_cat_ decreased by factor 5, suggesting a strong non-competitive inhibition. In contrast to Leu, its precursor KIC is a competitive inhibitor, targeting the substrate binding pocket of LeuA1 (Figure 6I and Figure S10). KIC shares structural similarities to the IPMS substrate KIV and raised the *K*_M_ by factor 2.3 at a *K*_Ι_ of 293.31 µM. In summary, both Leu and its α-keto acid KIC inhibits LeuA1 by a non-competitive and competitive mode of action, respectively (Figure 7).

*Regulation by propionyl*-*CoA*. Except Ac-CoA, none of the other acyl-CoA donors (Prop-CoA or Mal-CoA) are accepted as cosubstrates (Figure S7). This contrasts the KIV-dependend acyl-CoA hydrolytic activity from IPMS of other species. The phylogenetically related IPMS from the bacterium *Cupriavidus necator* (Figure 2) binds and converts Ac-, Prop- and Mal-CoA with a near similar velocity of 0.25–1 min^−1^ [45]. In *M. alpina,* Prop-CoA, but not Mal-CoA, inhibits the α-IPM synthesis by 43% at 200 µM (Figure 6J). Prop-CoA is a mixed-type inhibitor as it both increases *K*_Μ_ and decreases *k*_cat_ values (Figure 6K and Figure S10), as suggested for an inihibitor, binding both the free enzyme and the enzyme-substrate complex. The IMPS-substrate KIV is a branch point between Val and Leu biosynthesis in *M. alpina*. A lack of Val in the cell is probably sensed by its accumulating catabolic product Prop-CoA, which inhibits the KIV-consuming LeuA1, resulting in sufficient intracellular KIV to regenerate Val via BatA (Figure 7).

## 4. Discussion

Our data indicated a different mode of BCAA regulation in basal fungi dissimilar to other fungal divisions. The transcriptional regulation of BCAA biosynthesis is much less abundant in *M. alpina*, when compared to Dikarya. The absence of a common transcriptional regulator similar to Leu3 in *S. cerevisiae* [8] or LeuB in *A. nidulans* [15], suggests a different mode of regulation. Indeed, since Leu is an important building block for protein biosynthesis, PUFA production, and secondary metabolite biosynthesis in *M. alpina*, a constant Leu turnover is required.

Leu represses the expression of the malpinin synthetase gene and access of BCAAs impairs malpinin production. On first sight, this appears as an inverse regulation, since BCAAs are required of malpinin biosynthesis as well. However, an access of secondary metabolites may lead to a self-intoxication and is usually circumvented by a down-regulated expression, as shown for the gliotoxin gene cluster [46]. Many ascomycete SM gene clusters are regulated by the presence of a single specific amino acid, e.g., the isoflavipucine, the terrein gene cluster in *Aspergillus terreus*, or the aflatoxin biosynthesis in *Aspergillus flavus*, which are regulated by asparagine, methionine, or glutamine, respectively [24,47,48]. In *Fusarium graminearum*, the trichothecene transcriptional activator Tri6 is both a regulator for mycotoxin production and BCAA degradation, showing that primary and secondary metabolism are interwoven [49]. Fungal SM genes are hierarchically regulated by global transcription factors such as the nitrogen catabolite repressor AreA [50]. However, appropriate candidate proteins have not been identified in basal fungi yet [51].

In contrast to the malpinin biosynthesis, the genes of BCAA biosynthesis are marginally regulated by altered expression. Dikarya usually rely on both a transcriptional and metabolic BCAA regulation [1]. The BCAA-mediated down-regulation of BCAA biosynthesis genes have solely been determined for *leuA1* and *leuA2*, i.e., the first two genes in the late-stage phase of Leu biosynthesis. Transcriptional regulation of its homologs in ascomycetes rely on the zinc knuckle transcription factor Leu3/LeuB [52], which binds to upstream activating sequences (UASLEU) in promotor regions of at least five BCAA biosynthesis genes [53]. However, a *leu3* homolog is missing in *M. alpina*, and a merely constitutive expression of Leu biosynthesis is observed.

The *batA* gene, encoding the sole BCAA aminotransferase, is constitutively expressed like a house-keeping gene and is required for both BCAA anabolism and catabolism. This contrasts the regulation in ascomycetes or plants, in which at least two, and up to six, individual BATs are partially inversely transcriptionally regulated and, hence, are responsible for either BCAA biosynthesis or degradation. Human and yeast BATs form homodimers [54,55], while BatA is a monomeric enzyme at least in the tested conditions. However, it cannot be excluded that BatA may interact with other proteins to form a heterodimeric complex in vivo for a more fine-tuned functional modulation. Indeed, signalling kinases, such as protein kinase C, bind and phosphorylate human BATs and shift their function from a transaminase to a chaperone [56]. The removal of the N-terminal mitochondrial signal sequence (MIS) of BatA by mitochondrial processing peptidases [57] is required for the full functionality. This compartmentation is essential for *M. alpina* to regulate the metabolic flow of BCAAs. Indeed, mitochondrial compartmentation of BatA circumvents a competition with the cytosolic IPMS, LeuA1, for its common substrate KIV (Figure 7).

In contrast to BatA, IPMS is the major regulatory key enzyme in Leu and Val biosynthesis in *M. alpina* (Figure 7). Phylogenetically, the *leuA1* gene is most likely originated by HGT from a photosynthetic ancestor such as a cyanobacterium, whilst the Dikarya may have evolved an independent IPMS version [4]. Indeed, HGT from bacteria is the major driver for metabolic diversity in Mucoromycota [23]. IPMS plays a critical role and interconnects BCAA biosynthesis, BCAA degradation, β-oxidation, PUFA production, and secondary metabolite biosynthesis (Figure 7). LeuA1 is feedback-regulated by both Leu and its precursor KIC. These regulatory feedback loops are a common superordinate strategy to modulate enzyme activity across fungal kingdoms. Indeed, the IPMS of the basal fungus *P. blakesleeanus* can—at least in part—functionally replace the IPMS in yeast [4,5]. An allosteric inhibition of IPMS activity by Leu has been assigned to a 39 amino acid long, C-terminal regulatory region, identified by site mutation in *S. cerevisiae* [58] and confirmed by crystal-structure analysis of the *M. tuberculosis* IPMS [44]. A similar hydrophobic binding pocket (positions 450–482) is found in *M. alpina* LeuA1. In contrast to Leu, KIC is clearly a competitive inhibitor competing with KIV for the substrate binding pocket. The conversion of KIC to Leu by mitochondrial BatA requires a back-flow of cytosolic KIC into the mitochondria via monocarboxylate transporters [59], and KIC may accumulate in the cytosol. Hence, high cytosolic KIC:KIV ratios are sensed by LeuA1 and represses Leu biosynthesis prior to mitochondrial Leu formation. The third LeuA1 inhibitor is Prop-CoA. Prop-CoA is a potent inhibitor for various CoA-dependent C-transferases, including the pyruvate dehydrogenase, succinyl-CoA synthetase, ATP citrate lyase, and polyketide synthases in secondary metabolism [60,61]. Prop-CoA is a by-product of the β-oxidation of odd-chain fatty acids and is an end-product of amino acid degradation, including Val, Ile, Met, and Thr [61]. Hence, LeuA1 may sense the Val and Ile concentration in the cell (Figure 7); in case of a lack of Ile or Val due to enhanced degradation, its catabolite Prop-CoA can disable LeuA1 and, hence, block the KIV-consuming Leu biosynthesis. In turn, elevated KIV levels can replenish the intracellular Val pool by a BatA-mediated reaction.

## 5. Conclusions

In contrast to higher fungi, the BCAA biosynthesis pathways in basal fungi are predominantly metabolically, rather than transcriptionally, regulated. BCAA production is a constitutive process, as it is required for protein biosynthesis, fatty acid metabolism, and secondary metabolite production as well. However, it can be quickly modulated if environmental metabolite fluxes change. The main regulatory protein is the plant-derived α-isopropyl malate synthase, whose gene has been presumably introduced by horizontal gene transfer. The intimin interaction of basal fungi with bacteria or plants is a widely described phenomenon. Interspecific gene transfer provides an efficient strategy to adapt to altered environmental conditions and may have contributed to the ubiquitous spread of basal fungi in various ecological niches. From an industrial angle, the described complex inhibition loops regulating the leucine metabolism in *M. alpina* may help to increase the production yields of polyunsaturated fatty acids.

## Data Availability

All data and statistical analysis reported in this manuscript are listed in the Supplementary Materials.

## Supplementary Materials

The following materials available online at https://www.mdpi.com/article/10.3390/jof8020196/s1, Experimental procedures, Table S1: Oligonucleotides, Table S2: Plasmids, Table S3: UHPLC methods, Table S4: Amino acid sequences used for phylogenetic analysis of LeuA1 homologs, Table S5: Statistical analysis on the malpinin quantification in dependence of BCAA supply, Table S6: Gene expression data of *malA* and BCAA biosynthetic genes in *M. alpina*, Table S7: Statistical analysis of gene expression data, Table S8: Amino acid sequences used for phylogenetic analysis of BCAA aminotransferase homologs, Figure S1: Phylogenetic analysis of BCAA aminotransferases (BAT´s), Figure S2: SDS polyacrylamide gel electrophoresis (SDS-PAGE) of His_6_-tagged *M. alpina* enzymes, Figure S3: Size exclusion chromatography (SEC) of His_6_-tagged proteins, Figure S4: Impact of temperature and pH on BatA activity, Figure S5: Activity of BatA-L, BatA-S, and BatC, Figure S6: Impact of temperature and pH on LeuA1 activity, Figure S7: Acyl-CoA dependence of LeuA1 activity, Figure S8: Sodium and potassium dependence of LeuA1 activity, Figure S9: Impact of divalent cations on LeuA1 activity, Figure S10: Regulation of LeuA1 by primary metabolites, References. References [2,3,4,6,26,29,30,38,40,44,55,62,63,64,65,66,67,68,69,70,71,72,73,74,75] are cited in the supplementary materials.
