# Supplementary material for: Regulation of the Leucine Metabolism in Mortierella alpina
Source: J Fungi (Basel). 2022 Feb 18;8(2):196. doi: 10.3390/jof8020196 (PMC8880518; doi:10.3390/jof8020196)
Supplement: Supplementary file 1 [file jof-08-00196-s001.zip › jof-1594910-supplementary.pdf]

# Regulation of the leucine metabolism in *Mortierella alpina*

**Robin Sonnabend [1], Lucas Seiler [1] and Markus Gressler [1,*]**

[1] Pharmaceutical Microbiology, Friedrich-Schiller-University Jena, Leibniz Institute for Natural Product Research and Infection Biology - Hans-Knöll-Institute, Winzerlaer Strasse 2, 07745 Jena, Germany.
* Correspondence: markus.gressler@leibniz-hki.de

## Table of content

**Experimental procedures....2**

**Table S1. Oligonucleotides....6**

**Table S2. Plasmids ....9**

**Table S3. UHPLC methods ....10**

**Table S4. Amino acid sequences used for phylogenetic analysis of LeuA1 homologs....11**

**Table S5. Statistical analysis on the malpinin quantification in dependence of BCAA supply....13**

**Table S6. Gene expression data of *malA* and BCAA biosynthetic genes in *M. alpina*....14**

**Table S7. Statistical analysis of gene expression data....16**

**Table S8. Amino acid sequences used for phylogenetic analysis of BCAA aminotransferase homologs....20**

**Figure S1. Phylogenetic analysis of BCAA aminotransferases (BAT´s)....21**

**Figure S2. SDS polyacrylamide gel electrophoresis (SDS-PAGE) of $His_6$-tagged *M. alpina* enzymes....22**

**Figure S3. Size exclusion chromatography (SEC) of $His_6$-tagged proteins....23**

**Figure S4. Impact of temperature and pH on BatA activity....24**

**Figure S5. Activity of BatA-L, BatA-S, and BatC....25**

**Figure S6. Impact of temperature and pH on LeuA1 activity....26**

**Figure S7. Acyl-CoA dependence of LeuA1 activity....27**

**Figure S8. Sodium and potassium dependence of LeuA1 activity....28**

**Figure S9. Impact of divalent cations on LeuA1 activity ....29**

**Figure S10. Regulation of LeuA1 by primary metabolites....30**

**References....31**

# Experimental procedures

**Construction of plasmids for heterologous expression of *batA-L, batA-S, batC* and *leuA1*.**

Coding sequences for the two versions of *batA (batA-L* and *batA-S,* with and without the codons for the mitochondrial import signal, respectively*), batC* and *leuA1* were amplified from cDNA of *M. alpina* ATCC32222 (grown on AMM for 5 days). Oligonucleotides oRS37/oRS039 (*batA-L*), oRS038/oRS039 *(batA-S*), oRS049/oRS046 (*batC*) and oRS40/oRS041 (*leuA1*) (Table S1), respectively, and Phusion DNA polymerase (Thermo Fisher) were used. The PCR protocol was: 98°C, 2 min; 98°C, 15 s, 60°C, 15 s; 72°C, 60 s; 35 cycles; terminal hold at 72°C, 5 min. The DNA fragments were ligated into pJET1.2 using the CloneJET PCR cloning kit (Thermo Fisher). The genes *batA-L* and *batA-S* were excised with *Nde*I/*Hind*III and ligated into the accordingly digested pET28a(+) vector to create plasmids pRS04 and pRS05, respectively. Similarly, *batC* and *leuA1* were excised with *Nhe*I/*Hind*III and ligated into the *Nhe*I/*Hind*III-linearized pJET28a(+) vector (final plasmids pRS08 and pRS06). These expression plasmids were used to transform *Escherichia coli* BL21, using 50 µg $mL^{-1}$ kanamycin as selection marker.

**Heterologous production of enyzmes in *Escherichia coli* and size exclusion chromatography (SEC).**

Production of the N-terminally $His_6$-tagged proteins was performed in *Escherichia coli* BL21 in 400 mL 2xYT medium (20 g $L^{-1}$ tryptone, 10 g $L^{-1}$ yeast extract, 10 g $L^{-1}$ NaCl), supplemented with 50 mg $mL^{-1}$ kanamycin, at 37°C and 180 rpm. At an $OD_{600}$ of 0.6–0.8, the temperature was shifted to 16°C, and gene expression was induced by addition of 1 mM isopropyl-β-D-1-thiogalactopyranoside (IPTG). After 16 h, cells were harvested by centrifugation and the pellet was resuspended in binding buffer (50 mM NaH2PO4, 300 mM NaCl, 10 mM imidazole, pH 8.0). After cell lysis by sonification, the lysate was centrifuged, and the cell-free supernatant was transferred to an equilibrated gravity affinity column with 1 mL Protino $Ni^{2+}$-NTA resin (Macherey-Nagel). After 25 min incubation on ice, the resin was washed with binding buffer containing increasing concentrations of imidazole (10, 25 and 50 mM). Pure proteins were eluted with binding buffer with 250 mM imidazole. The enzymes were re-buffered in 50

mM Tris-HCl (pH 8.0) by gel filtration (PD10, Cytiva). Protein concentration was determined using the Pierce BCA Protein Assay Kit (Thermo Fisher) versus a serial dilution of the provided bovine serum albumine (BSA) standard solution. After supplementation with 10% glycerol, enzymes were stored at -80°C without loss of activity for up to 4 months. For determination of the oligomeric state by size exclusion chromatography (SEC), the native and denatured (95°C, 5 min) enzymes were re-buffered in SEC buffer (10 mM $NaH_2PO_4$, 140 mM NaCl, pH 7.5) and applied to a Superdex 200 increase 10/300GL column (Cytiva) on an FPLC Äkta Pure 25 instrument using an isocratic run. Commercial protein standards served as size reference (gel filtration HMW and LMW calibration kits, Cytiva).

**Chromatographical quantification of α-amino acids and α-keto acids**

To determine the intracellular amino acids, the fungus was grown in AMM with or without 10 mM of one of the BCAAs (Ile, Leu, Val) or 10 mM of each BCAA. Mycelium was harvested using Miracloth (Merck Millipore), extensively washed with 200 mL tap water and lyophilized. The dry fungal mass (50-400 mg) was ground to a fine powder with a micropistill and extracted twice with 100 μL methanol per 100 mg powder for 10 min each. After brief centrifugation (14,000 x g, 10 min), 1 μL thereof was applied to a SeQuant ZIC-HILIC column (Merck) using method A or method B (Table S3). The 20 standard L-amino acids and six α–keto acids (α-ketoisovalerate (KIV), α-ketomethylvalerate (KMV), α-ketocaproate (KIC), α-ketobutyrate (KB), α-ketoglutarate (KG), pyruvate) served as references. BAT activity was determined based on a calibration curve with L-glutamate monitored in a range of 0.01 – 10 mM using the extracted ion chromatogram (EIC) in positive mode.

**Chromatographical quantification of malpinins**

*M. alpina* was cultivated in 50 mL AMM without or with 10 mM of one of the BCAAs or 10 mM of each BCAA (Ile, Val, Leu). The fungal total fungal biomass was collected with Miracloth (Merck, Millipore), lyophilized and the dry biomass was weighted. The cell-free supernatant (50 mL) was adjusted to pH 6.5 by 4 M HCl and extracted twice with an equal volume of ethyl acetate. The solvent was removed by a rotary evaporator and the residue was dissolved in 2 mL methanol. After brief centrifugation (10,000

x g, 10 min), 10 μL of the crude extract was applied to a Zorbax Eclipse Plus C18 RRHD (Agilent) column using HPLC method C (Table S3). Metabolites were quantified from the extracted ion chromatograms (EIC) monitored in the positive detection mode. For quantification, a calibration curve of malpinin A (m/z 859 $[M+H]^+$) ranging from 0 to 500 μg/mL served as reference.

**Phylogenetic analysis of BCAA aminotransferases and α-isopropylmalate synthases.**

The evolutionary history was inferred by using the Maximum Likelihood method and Le_Gascuel_2008 model [1]. The bootstrap consensus tree inferred from 1000 replicates [2]. Evolutionary analyses were conducted in MEGA11 [3].

**Statistical analysis**

Statistical analyses were performed using the Dunnet-Test (One-Way and Two-Way-Anova) implemented in the GraphPad Prism 7.04 Software package. Significant values are denoted as: ns, not significant, * p<0.05, **p<0.01, *** p<0.001 and **** p<0.0001.

**cDNA sequences**

Underlined sequences indicate codons encoding the mitochondrial import sequence.

**>*batA* (1224 bp)**

ATGTTTGTCCAAAGAACACTCCGCCAGGCTGTCAAGGCCCACTCCGCCTTCGCCTCCTCGCCC
GCCGCCTACAGCGCCAAGGTCCTCTCCTCGCGCTTCTACAGCACCTCCATCGCGCCCCTCGAT
GCCTCCAAGCTCGAGATCAACGCCTCCAAGAACAAGAAGCCACTGCAGGAGAACTCGCAGC
TGGTCTTTGGCAAGACCTTCTCCGACAACATGCTCACCATCGAGTGGGAGGCCGGCAAGGGCT
GGGCCAAGCCCGAGATCAAGGAGTATGGCAAGCTCCACCTCGACCCCTCCGCGGTCGTCTTC
CACTACTCGTTCGAGTGCTTCGAGGGCATGAAGGCCTACAAGGACAAGCACGGCAAGACCCG
TCTCTTCCGTCCCGACATGAACATGAAGCGTTTCAACAGGAGTGCTGCCCGCATTGCCCTTCC
CAACTTTGAGGACGAGGAGTTGATCAAGTTGATTGGCGAGTTCTTGAAGGTCGATGACCGCTG
GATCCCCAAGGGCCGTGGTTACTCGCTCTACCTCCGTCCCACCATGATCGGCACCCAAGAGTC
CCTGGGAGTGGGTGCATCGAACAAGGCCTTGATCTTTGTCATTGCCTCGCCTGTCGGTCCTTAC
TTCCCCTCGGGATTCAATGCCGTCAACCTGTTGGCGACCTCGGACAAGGTCCGTGCCTGGCCC
GGTGGAACGGGTGATGCCAAGGTCGGAGGCAACTATGCGCCTTGCATCAAGCCCCAGTTGGA
TGCCAACAAGGAGGGTTACCAGCAGAACCTGTGGCTGTTTGGCGCTGACCACCAGGTCACCG
AGGTCGGCACCATGAACTGCTTTGTCTTCTGGAAGAACGAGCAAGGAGAGAGAGAGCTGGTC
ACCCCTGACCTGGATGGCTCCATCCTGCCCGGAGTCACCCGTGACAGTATCCTGAGCCTTGCC
CGTCAATGGGGCGAGTTCAAGGTCTCGGAGCGCAAGTTCACCATGGCCGATCTCGTCAAGGC
TGATAAGGAAGGCCGTATCATTGAAATGTTTGGTGCTGGAACGGCTTGCATTGTCTGCCCTAT
CAAGAAGATCCACTTTGAGGGTAAGGACATCAACATCCCCCTGGATCCCACTGACAAGACCA
GCCAGGCTGGCAAGTTGACCAAGCGCATCAACGATGCCATCATGGACATCCAGTACGGTGAT
GTCGAGGGTCCCAAGGGCTGGTCCGTCGTTGTCTAA

**>*batC* (1104 bp)**

ATGACCTGCACACTGCCACCACCCCCAACCTCCGCCATCGACTGGGACAACCTCGGCTTCAA
GTGGGTCGACACCAACGGGCACGTCAAGTACATCCACAAGGACGGCCAGTGGGACCAGGGC
GAGTTTGTCCGCGACTCGTACATCAAGATGCACGTCTGTGCCCCAGCACTGAACTATGGCCAG
GAGTGCTTCGAGGGCATCAAGGCGTTCAGGTGCAAGGACGGCCAGGTCCGTATCTTCCGCCC
CGATGCCAACGCCAAGCGTATGCGCTACTCTGCAGACCTGGCCTGCATGGCCGCCGTGCCCG
AGGATCTTTTTGTGCAGGCATGCGAGCGCGCTGTGGCCAACAATCTCGAGTTTGTGCCCCCCT
ACGGCTCTGGCGGGGCCCTCTATCTGCGCCCTCTCCTCCTTGGCACTGGCCCCGAGATTGGCCT
TTATCCCGCTCCAGAGTTCACGTTCATTGTCATGGTCATTCCCGTTGGCAACTTTTACAAGTCC
GGAGTAAAGCCAGTGGACTGCCTTGTCGTTGACAACTTTGACCGAGCGGCGCCCCTGGGAAC
TGGAGCGGCTAAACTGGGTGGCAACTACGCGCCGGTTCTGAAGCACAGCAGCGCGGCCAAG
GAGCAGGGATACGCTATCACACTCCACCTCGACTCCAAGACACACTCGAACGTGGATGAATT
CAGCACTTCCAACTTTGTGGCCCTCACCTACCCCTCCGCCGAGCACGACAACAAGCCCGTCTT
TTGCGCCCCCGAAAGCCCCTCCATCCTCAACTCTGTCACCAAGCAATCACTGGTTCAGCTCGC
CAAAAGTTTTGGCTGGGCCGTTGAAAACAGCGTCGTGCCCTTCACTGACATCACAAGCAATCG
TTTCGAGGAAATTGCTGCCTGCGGGACTGCAGCGGTCATCACGCCTGTCAAATCCATCGAACG
TCAGGGAACAAAGACTCTTATCGGCACTTCCGCCACTCAGGAGGCCATTGGTGCCGGATTTCT
CCGCCTCTTTACAGCTATGAGGGGCATCCAGAATGGCGATCTTGAGGATCCATATGGTTGGAT
GCAGCCTGCCCAAGGCATTGCCCCTCGGCAGGAGGCGTAA

**>*leuA1* (1689 bp)**

ATGGCACAAGCAAATAAAGACAAGTTGATCATTTTCGACACTACTCTCAGAGATGGCGAGCA
GTCCCCAGGAGTAACCCTTACCGTGAACGAAAAGATTGAAATCGCTAAGCAGCTCTCTCGCCT
CGGAGTTGATGTCCTCGAGGCTGGTTTCCCTGTGGCATCCCAGGGTGACTTTGATGCCGTTGAG
CGGATTGCGACTGAGGTTGGCCCCCTCATGGTCGGCCGTGAAGCCCTTGGCAAGCCCATTACA
ATTGCGGGTCTTGCTCGTGCCGTGCCAGCAGATATCCAGCGATGCTACGATGCCATCGCAAAG
GCCCCTCACAAACGCATTCACACCTTCCTCGCTACCTCGGACATCCACCTCGAGCACAAACTC
AAAATTTCGCGCGACGAATGCGTAAAGCGCGCAGTGGCCGCAGTATCCTTCGCCAAGACCTT
GGTCCAGGACGTCGAGTTTTCGCCTGAGGATGCAGGAAGGAGCGACCAGGACTTTTTGTGCA
AGGTCCTCTCTGCAGTAATCGAGGCCGGAGCAACAACTCTCAACATCCCTGACACAGTAGGC
TACAACATGCCGGAGGAGTATGGGTCAATGATCAAGTACTTGATCGCCAATACGAAGGGTTC
GGACACGGTCATCTGGTCTGCTCACTGCCACAACGACCTCGGACTTGCCACTGCCAACACCCT
CTCGGGCATCATGAACGGAATTCGCCAGGTTGAGGTCACCATCAATGGTATCGGCGAGCGTG
CGGGAAATACTGCCATGGAGGAGGTCGTCATGGCCATCCACACCCACCCCAACTTTTTCCCTG
TCTACCATACCATCAACACGCCCCTGTTCTTCAGGATCTCACAGCTCGTGTCATCGCTCTCGGG
AATGGCAGTACAATCTAACAAGGCCATTGTAGGGGCCAACGCATTCCTGCACGAGTCTGGTA
TTCATCAGGATGGTGTTCTTAAGAATAAGACCACATATGAGATCATCAGCCCCGAGACAGTA
GGTGCCAACACTGTCAACCTTGTCCTTGGCAAGCATTCTGGACGTGCTGCTTTCAAGTCTCGTC
TCCAGGAGCTTGGTTTTGGTGAGCTGACAGACGAAGAGTTCCAGGTGGCGTTCAACAGTTTCA
AGACCTTGGCCGATAGCAAGAAGCGTGTGACCGAGCAGGATTTGCTTGCCTTGCTGTCGGATC
AGGTCTCGGCCGCGTCCGGGGACAAGGCCACGTTCGTGATCAAGTCGCTCCAGGTGGTCTCG
GGCACATCGTTTGCAACCGCCACGGTCCAGTTGCAGAACAGCCAGACCGGCGATGTGGTGGT
GGATGCTGCCATTGGCAAGTCTGGACCAGTCGAGGCCGTCTTTTTGGCGATCCAGCGCTTGGT
GGGCAAGAGTATTCGCCTGCTCAAGTTTGATATCACGGCCGTAGGTGAGGGTGTCGATGCTTT
GGGCCAGGTTTCAGTCAAGATTGCAGAGGACACGTCGTCACCCGGTTCCTCGGACAGTGATG
AAAATGCAGAGGAGGCAAAGGACAATGAGAGCAAGGCCAATGGAATCAGCCAGGCCACGT
ACCACGGTCACGGTGCCGACTCGGATATTATCCAGGCTGCGACCAAAGCCTACCTCAATGCC
ATTAACCGCCTGGTAGCAAATGAGAACGAGCGTCTTCTCGGTGAACGCAAGGTCGATGTTTA
A

**Table S1. Oligonucleotides.**

| oligonucleotide | 5´-3´sequence | target gene | purpose | fragment size (gDNA) [bp] | fragment size (cDNA) [bp] | Primer efficiency or restriction site |
|---|---|---|---|---|---|---|
| oRS001 | CGGTACCAGCATGTTTGTGG | *atmA* | expression analysis (qRT-pCR) | 242 | 119 | 0.97 |
| oRS002 | CCTTCTTGAGCAACTCTTCG | | expression analysis (qRT-pCR) | | | |
| oRS005 | GGCCTACAAGGACAAGCACG | *batA* | expression analysis (qRT-pCR) | 315 | 182 | 0.93 |
| oRS006 | CGAGTAACCACGGCCCTTG | | expression analysis (qRT-pCR) | | | |
| oRS007 | CAGAGTTCACGTTCATTGTCATGG | *batC* | expression analysis (qRT-pCR) | 324 | 193 | 0.96 |
| oRS008 | GCGTATCCCTGCTCCTTGG | | expression analysis (qRT-pCR) | | | |
| oRS009 | GCAGCAGTCAAGGTCGTTGG | *ilvA* | expression analysis (qRT-pCR) | 281 | 194 | 0.94 |
| oRS010 | GTTAGGTTGTGCTCCCGGAG | | expression analysis (qRT-pCR) | | | |
| oRS011 | GGTTTCTCCGATGGCTGATC | *ilvB* | expression analysis (qRT-pCR) | 253 | 142 | 0.97 |
| oRS012 | GAACACGACCATGGGAGTTCC | | expression analysis (qRT-pCR) | | | |
| oRS013 | GAACCATCACCGAGACTCAGC | *ilvC* | expression analysis (qRT-pCR) | 296 | 192 | 0.96 |
| oRS014 | CCTTTCCAGCATCAAGACACTCC | | expression analysis (qRT-pCR) | | | |
| oRS015 | TCGCTCCTTCTCGGTCATGG | *ilvE1* | expression analysis (qRT-pCR) | 281 | 186 | 0.96 |
| oRS016 | GTGTCGTTCTTGAAGGTCTCC | | expression analysis (qRT-pCR) | | | |
| oRS017 | CTCGCTCTCCCTCTTGGCTC | *ilvE2* | expression analysis (qRT-pCR) | 286 | 176 | 0.97 |
| oRS018 | CCTTGAAGGTTTCCTGCAGC | | expression analysis (qRT-pCR) | | | |
| oRS019 | CGAGGTGGCTGATCTGAGTC | *ilvF1* | expression analysis (qRT-pCR) | 286 | 155 | 0.95 |
| oRS020 | GATCAACAGCAACTCGCGC | | expression analysis | | | |

| | | | | | | |
|---|---|---|---|---|---|---|
| | | | (qRT-pCR) | | | |
| oRS021 | GGATGACGGTGGTGATGAAGG | *ilvF2* | expression analysis (qRT-pCR) | 229 | 108 | 0,94 |
| oRS022 | CCTTGAACCCAGTGTAGTCCAAC | | expression analysis (qRT-pCR) | | | |
| oRS023 | TTGACCAAGAACGGCCAGC | *leuA2* | expression analysis (qRT-pCR) | 141 | 141 | 0,94 |
| oRS024 | GAACTGTTCACTGCCATCGG | | expression analysis (qRT-pCR) | | | |
| oRS025 | CTTCAAGACTGAGCTCATCGG | *leuB1* | expression analysis (qRT-pCR) | 389 | 139 | 0,92 |
| oRS026 | GTTTCCTGTCGCCCACTTGG | | expression analysis (qRT-pCR) | | | |
| oRS027 | GGTCGCCAAAGTCCGATCC | *leuB2* | expression analysis (qRT-pCR) | 402 | 153 | 0.93 |
| oRS028 | CTCCTACTGCTCCCAAGAGG | | expression analysis (qRT-pCR) | | | |
| oRS031 | GCTGGTTTCCCTGTGGCATCC | *leuA1* | expression analysis (qRT-pCR) | 376 | 135 | 0.94 |
| oRS032 | CGTAGCATCGCTGGATATCTGC | | expression analysis (qRT-pCR) | | | |
| oRS033 | AGATGAGACGCCCTATAGAAAGC | *leuE1* | expression analysis (qRT-pCR) | 258 | 122 | 0.99 |
| oRS034 | GTTCGTTGTAAATTTTGCGGACC | | expression analysis (qRT-pCR) | | | |
| oJW01 | CATCGATCTGGCCTACATGG | *gpdA* | expression analysis (qRT-pCR) | 229 | 80 | 1.04 |
| oJW02 | CCACCTTGCCCTTGTAGC | | expression analysis (qRT-pCR) | | | |
| oJW03 | GTATGTGCAAGGCCGGTTTCG | *actB* | expression analysis (qRT-pCR) | 224 | 100 | 1.00 |
| oJW04 | CCCATACCGACCATCACACC | | expression analysis (qRT-pCR) | | | |
| oJW175 | TACGAGGAGCCAAGAGGTG | *malA* | expression analysis (qRT-pCR) | 99 | 99 | 0.94 |
| oJW176 | GACAAAGAAGCCGTCGTTCC | | expression analysis (qRT-pCR) | | | |
| oRS037 | GTGCCGCGCGGCAGCCATATGATGTTTGTCCAAAGAACACTCCG | *batA-L* | Bat1L production in *E. coli* | 1824 | 1266 | *Nde*I |
| oRS038 | GTGCCGCGCGGCAGCCATATGAGCACCTCCATCGCGCCC | *batA-S* | Bat1S production in *E. coli* | 1763 | 1205 | *Nde*I |
| oRS039 | CTCGAGTGCGGCCGCAAGCTTTTAGACAACGACGGACCAGC | *batA* | Bat1L/S production in *E. coli* | | | *Hind*III |

| | | | | | | |
|---|---|---|---|---|---|---|
| oRS049 | CGCGGCAGCCATATGGCTAGCATGACCTGCACACTGCCACC | *batC* | BatC production in *E. coli* | 1344 | 1104 | *Nhe*I |
| oRS046 | GCTCGAGTGCGGCCGCAAGCTTACGCCTCCTGCCGAGGG | *batC* | BatC production in *E. coli* | | | *Hind*III |
| oRS040 | CGCGGCAGCCATATGGCTAGCATGGCACAAGCAAATAAAGACAAG | *leuA1* | LeuA1 production in *E. coli* | 2126 | 1775 | *Nhe*I |
| oRS041 | CTCGAGTGCGGCCGCAAGCTTTTAAACATCGACCTTGCGTTCAC | *leuA1* | LeuA1 production *in E. coli* | | | *Hind*III |

**Table S2. Plasmids.**

| Plasmid name | vector backbone | expressed gene | position of His6-tag | reference |
|---|---|---|---|---|
| pRS02 | pET28a(+) | *batA-L* | N-terminus | This study. |
| pRS04 | pET28a(+) | *batA-S* | N-terminus | This study. |
| pRS06 | pET28a(+) | *leuA1* | N-terminus | This study. |
| pRS08 | pET28a(+) | *batC* | N-terminus | This study. |

**Table S3. UHPLC methods.**

| | method A | method B | method C |
|---|---|---|---|
| **purpose** | amino and keto acid quantification | amino and keto acid quantification | malpinin quantification |
| **instrument** | Agilent 1290 infinity II UHPLC | Agilent 1290 infinity II UHPLC | Agilent 1290 infinity II UHPLC |
| **solvent A** | 20 mM $NH_4CH_3COO$, pH 6.8 | 20 mM $NH_4CH_3COO$, pH 6.8 | water + 0.1% FA |
| **solvent B** | acetonitrile | acetonitrile | acetonitrile |
| **gradient** | 0-13 min: 90-57% B,<br>13-15 min: 57-70% B,<br>15-16 min: 30% | 0-12.5 min: 90-60% B,<br>12.5-15 min: 60-40% B,<br>15-16 min: 40% B | 0-4 min: 5-72% B,<br>4-4.5 min: 72-95% B,<br>4.5-5 min: 95% B |
| **temperature** | 45°C | 45°C | 30°C |
| **flow** | 0.4 mL $min^{-1}$ | 0.4 mL $min^{-1}$ | 1 mL $min^{-1}$ |
| **column** | SeQuant®ZIC®-HILIC 3.5 µm, 100 Å | SeQuant®ZIC®-HILIC 3.5 µm, 100 Å | Zorbax Eclipse Plus C18 RRHD (Agilent) |
| **column dimension** | 250 x 2.1 mm, 3.5 µm | 150 x 2.1 mm, 3.5 µm | 50 mm × 2.1 mm, 1.8 µm |
| **detection** | Agilent 6130 single quadrupole MS, ESI ionization source | Agilent 6130 single quadrupole MS, ESI ionization source | Agilent 6130 single quadrupole MS, ESI ionization source |

**Table S4. Amino acid sequences used for phylogenetic analysis of LeuA1 homologs.**

| Organism | Protein | Accession number | reference |
|---|---|---|---|
| *Anaeromyces robustus* | | NP_177544.1 | |
| *Arabidopsis thaliana* | IPMS1 | NP_197693.1 | [4] |
| *Arabidopsis thaliana* | IPMS2 | ORX84989.1 | [4] |
| *Aspergillus fumigatus* | LeuC | EDP56737.1 | [5] |
| *Aspergillus nidulans* | LeuA | XP_658444.1 | [6] |
| *Azotobacter vinelandii* | | ZP_00088628.1 | |
| *Bacillus cereus* | LeuA | ZP_03238480.1 | |
| *Bacterial endosymbiont of Mortierella elongata* FMR23-6 | | GAM51972.1 | |
| *Bacteroides fragilis* | aIPMS | KXU51314.1 | [7] |
| *Basidiobolus meristosporus* | | ORX90720.1 | |
| *Blyttiomyces helicus* | | RKO85405.1 | |
| *Botrytis cinerea* | | XP_001553290.1 | |
| *Caulobacter vibrioides* | | WP_010919415.1 | |
| *Chytriomyces confervae* | | TPX76259.1 | |
| *Clostridium formicaceticum* | aIPMS | AOY76370.1 | [7] |
| *Clostridium kluyveri* | aIPMS | WP_012102499.1 | [7] |
| *Clostridium pasteurianum* | aIPMS | KRU14690.1 | [7] |
| *Corynebacterium glutamicum* | | P42455.1 | |
| *Cryptococcus neoformans* | | XP_572334.1 | |
| *Cupriavidus necator* | IPMS | WP_042884649.1 | [8] |
| *Deinococcus radiodurans* | | AAF11047.1 | |
| *Desulfovibrio carbinoliphilus* | | EHJ49596.1 | |
| *Escherichia coli* | | P09151.5 | |
| *Glycine max* | IPMS | Glyma.03G005700.1 | [9] |
| *Haemophilus influenzae* | | P43861.1 | |
| *Klebsiella pneumoniae* | | WP_008806146.1 | |
| *Laccaria bicolor* | | XP_001874900.1 | |
| *Linderina pennispora* | | ORX68852.1 | |
| *Linnemannia elongata* | | OAQ34495.1 | |
| *Methanocaldococcus jannaschii* | | WP_010870707.1 | |
| *Micromonas pusilla* | | EEH52949.1 | |
| *Moorella thermoacetica* | aIPMS | WP_011393738.1 | [7] |
| ***Mortierella alpina*** | **LeuA1** | **ADAG01001098** | **This study.** |
| *Mucor circinelloides* | | EPB85936.1 | |
| *Mycetohabitans rhizoxinica 1* | | WP_041754403.1 | |
| *Mycetohabitans rhizoxinica 2* | | WP_041753704.1 | |
| *Mycoavidus cysteinexigens* | | BBE09271.1 | |
| *Mycoavidus sp.* | | BBO59729.1 | |
| *Mycobacterium tuberculosis* | MtIPMS | NP_218227.3 | [10] |
| *Neisseria meningitidis* | aIPMS | WP_050184874.1 | [11] |
| *Neocallimastix californiae* | | ORY17376.1 | |
| *Neurospora crassa* | | XP_964875.1 | |
| *Phycomyces blakesleeanus* | LeuA1 | ADK54799.1 | [12] |
| *Physcomitrella patens* | LeuA1 | Pp3c3_21830V3.1 | |
| *Physcomitrella patens* | LeuA2 | Pp3c4_4420V3.1 | |
| *Piromyces finnis* | | ORX47300.1 | |
| *Podila verticillata* | | KFH70037.1 | |
| *Powellomyces hirtus* | | TPX55294.1 | |
| *Pseudomonas aeruginosa* | | WP_012075129.1 | |
| *Pyricularia oryzae* | LEU4 | XP_003716219.1 | [13] |
| *Pyricularia oryzae* | LEU9 | XP_003720428.1 | [13] |
| *Pyrococcus abyssi* | | WP_010868464.1 | |
| *Pyrococcus furiosus* | | AAL81061.1 | |
| *Ralstonia solanacearum* | | AOE89993.1 | |
| *Rhizoclosmatium globosum* | | ORY51790.1 | |

| | | | |
|---|---|---|---|
| *Rhizopus delemar* | | EIE92225.1 | |
| *Saccharomyces cerevisiae* | LEU4 | NP_014295.1 | [14] |
| *Saccharomyces cerevisiae* | LEU9 | NP_014751.1 | [15] |
| *Salmonella enterica ser. Typhimurium* | | P15875.4 | |
| *Schizosaccharomyces pombe* | Leu3 | O59736.2 | [12] |
| *Sinorhizobium meliloti* | | Q9X7L2.3 | |
| *Solanum pennellii 1* | | AAB61599.1 | |
| *Solanum pennellii 2* | | AAB61598.1 | |
| *Spinacia oleracea* | | XP_021839739.1 | |
| *Spizellomyces punctatus* | | XP_016611093.1 | |
| *Staphylococcus epidermidis* | | BBK81946.1 | |
| *Streptomyces coelicolor* | | O31046.2 | |
| *Synchytrium endobioticum* | | TPX34422.1 | |
| *Synchytrium microbalum* | | XP_031024433.1 | |
| *Synechococcus sp.* | | Q7U892.1 | |
| *Synechocystis spec.* | | WP_010873307.1 | |
| *Thalassiosira pseudonana* | | XP_002286323.1 | |
| *Thamnocephalis sphaerospora* | | RKP05169.1 | |
| *Thermosynechococcus elongatus* | | WP_011057237.1 | |
| *Trichodesmium erythraeum* | | Q112U2.1 | |
| *Ustilago maydis* | | KIS67652.1 | |
| *Yersinia pestis* | | NP_404175.1 | |

**Table S5. Statistical analysis on the malpinin quantification in dependence on BCAA supply.** Significance values are indicated as: ns, not significant; * p<0.05, ** p<0.01; *** p<0.001 and **** p<0.0001. Groups without significant changes are indicated with the same latter (a-d) in the compact letter display (Figure 3).

| Compared groups | Mean Diff. | 95.00% CI of diff. | Summary | P Value |
|---|---|---|---|---|
| **AMM 5d vs. AMM 7d** | 4.382 | 1.906 to 6.859 | *** | 0.0006 |
| **AMM 5d vs. AMM+ile 5d** | -1.714 | -4.19 to 0.7624 | ns | 0.2543 |
| **AMM 5d vs. AMM+ile 7d** | -3.941 | -6.417 to -1.464 | ** | 0.0017 |
| **AMM 5d vs. AMM+leu 5d** | -5.777 | -8.254 to -3.301 | **** | <0.0001 |
| **AMM 5d vs. AMM+leu 7d** | -2.892 | -5.368 to -0.4155 | * | 0.0192 |
| **AMM 5d vs. AMM+val 5d** | 0.4465 | -2.03 to 2.923 | ns | 0.9947 |
| **AMM 5d vs. AMM+val 7d** | 1.798 | -0.4174 to 4.013 | ns | 0.1398 |
| **AMM 5d vs. AMM+ile/leu/val 5d** | 5.655 | 3.44 to 7.87 | **** | <0.0001 |
| **AMM 5d vs. AMM+ile/leu/val 7d** | 7.947 | 5.732 to 10.16 | **** | <0.0001 |
| | | | | |
| **AMM 7d vs. AMM 5d** | -4.382 | -6.859 to -1.906 | *** | 0.0006 |
| **AMM 7d vs. AMM+ile 5d** | -6.096 | -8.809 to -3.383 | **** | <0.0001 |
| **AMM 7d vs. AMM+ile 7d** | -8.323 | -11.04 to -5.61 | **** | <0.0001 |
| **AMM 7d vs. AMM+leu 5d** | -10.16 | -12.87 to -7.447 | **** | <0.0001 |
| **AMM 7d vs. AMM+leu 7d** | -7.274 | -9.987 to -4.561 | **** | <0.0001 |
| **AMM 7d vs. AMM+val 5d** | -3.936 | -6.648 to -1.223 | ** | 0.0038 |
| **AMM 7d vs. AMM+val 7d** | -2.585 | -5.061 to -0.1082 | * | 0.039 |
| **AMM 7d vs. AMM+ile/leu/val 5d** | 1.273 | -1.204 to 3.749 | ns | 0.5425 |
| **AMM 7d vs. AMM+ile/leu/val 7d** | 3.565 | 1.088 to 6.041 | ** | 0.004 |
| | | | | |
| **AMM+ile 7d vs. AMM 5d** | 3.941 | 1.464 to 6.417 | ** | 0.0017 |
| **AMM+ile 7d vs. AMM 7d** | 8.323 | 5.61 to 11.04 | **** | <0.0001 |
| **AMM+ile 7d vs. AMM+ile 5d** | 2.227 | -0.486 to 4.94 | ns | 0.1331 |
| **AMM+ile 7d vs. AMM+leu 5d** | -1.837 | -4.549 to 0.8761 | ns | 0.2732 |
| **AMM+ile 7d vs. AMM+leu 7d** | 1.049 | -1.664 to 3.762 | ns | 0.7964 |
| **AMM+ile 7d vs. AMM+val 5d** | 4.387 | 1.675 to 7.1 | ** | 0.0015 |
| **AMM+ile 7d vs. AMM+val 7d** | 5.738 | 3.262 to 8.215 | **** | <0.0001 |
| **AMM+ile 7d vs. AMM+ile/leu/val 5d** | 9.596 | 7.119 to 12.07 | **** | <0.0001 |
| **AMM+ile 7d vs. AMM+ile/leu/val 7d** | 11.89 | 9.411 to 14.36 | **** | <0.0001 |

**Table S6. Gene expression data of *malA* and BCAA biosynthetic genes in *M. alpina*.** $C_t$ values of each set of 6 individual replicates are displayed.

| gene | AMM 5d | | | | | | AMM 7d | | | | | |
|---|---|---|---|---|---|---|---|---|---|---|---|---|
| ***gpdA*** | 14.57 | 13.86 | 14.16 | 14.18 | 14.94 | 14.26 | 16.32 | 14.95 | 15.19 | 16.57 | 14.28 | 15.52 |
| ***actB*** | 17.23 | 16.12 | 16.77 | 17.26 | 17.39 | 16.86 | 18.08 | 17.60 | 17.60 | 18.20 | 17.20 | 17.41 |
| ***malA*** | 18.04 | 18.35 | 17.10 | 17.95 | 18.44 | 17.09 | 18.60 | 18.58 | 18.27 | 18.97 | 18.42 | 18.44 |
| ***ilvA*** | 22.02 | 20.91 | 22.00 | 21.64 | 21.82 | 22.05 | 23.12 | 21.79 | 22.08 | 23.27 | 21.57 | 22.56 |
| ***ilvB*** | 24.52 | 23.11 | 23.55 | 25.02 | 23.63 | 24.05 | 25.83 | 23.82 | 23.81 | 25.96 | 24.00 | 24.16 |
| ***ilvC*** | 18.71 | 18.47 | 18.21 | 18.91 | 18.31 | 19.01 | 19.36 | 20.03 | 19.73 | 19.67 | 19.59 | 19.41 |
| ***ilvE1*** | 17.72 | 18.05 | 17.33 | 17.57 | 18.00 | 17.43 | 20.29 | 18.17 | 18.76 | 20.29 | 18.45 | 18.59 |
| ***ilvE2*** | 17.30 | 17.70 | 17.37 | 17.35 | 16.88 | 17.34 | 19.02 | 18.49 | 18.14 | 19.21 | 18.15 | 18.18 |
| ***ilvF1*** | 20.23 | 19.80 | 20.41 | 20.24 | 19.46 | 20.29 | 22.33 | 20.58 | 19.82 | 22.00 | 20.40 | 19.75 |
| ***ilvF6*** | 20.08 | 21.14 | 20.24 | 20.15 | 20.07 | 20.06 | 21.41 | 21.08 | 20.38 | 21.36 | 20.23 | 19.48 |
| ***leuA1*** | 19.05 | 18.38 | 18.83 | 18.83 | 18.54 | 18.75 | 19.84 | 19.53 | 19.56 | 18.93 | 19.26 | 19.24 |
| ***leuA2*** | 16.89 | 15.84 | 17.31 | 17.06 | 16.46 | 17.09 | 18.38 | 17.02 | 17.42 | 18.12 | 17.99 | 17.17 |
| ***leuB1*** | 17.63 | 18.01 | 17.87 | 18.06 | 16.97 | 17.47 | 18.75 | 18.30 | 18.44 | 19.25 | 18.32 | 18.07 |
| ***leuB2*** | 18.12 | 17.39 | 17.60 | 18.24 | 17.54 | 17.86 | 19.25 | 18.43 | 18.45 | 19.14 | 18.66 | 18.37 |
| ***batA*** | 18.69 | 18.47 | 18.46 | 18.67 | 18.08 | 18.53 | 19.65 | 18.95 | 19.06 | 19.40 | 18.85 | 19.03 |
| ***batC*** | 18.34 | 18.75 | 18.47 | 18.26 | 18.32 | 18.42 | 20.14 | 19.12 | 19.39 | 20.15 | 19.35 | 19.19 |

| gene | AMM + Ile 5d | | | | | | AMM + Ile 7d | | | | | |
|---|---|---|---|---|---|---|---|---|---|---|---|---|
| ***gpdA*** | 14.40 | 15.03 | 17.69 | 17.60 | 14.66 | 14.66 | 15.60 | 15.78 | 17.24 | 16.97 | 16.03 | 16.07 |
| ***actB*** | 17.07 | 17.14 | 20.04 | 19.67 | 17.33 | 17.68 | 17.78 | 18.02 | 18.63 | 18.64 | 17.58 | 17.64 |
| ***malA*** | 16.13 | 15.99 | 20.01 | 20.01 | 16.21 | 16.29 | 17.11 | 17.08 | 19.19 | 19.24 | 17.57 | 17.57 |
| ***ilvA*** | 19.76 | 19.56 | 22.15 | 22.05 | 20.53 | 20.87 | 20.23 | 20.42 | 21.01 | 20.88 | 20.41 | 20.36 |
| ***ilvB*** | 23.37 | 23.65 | 23.04 | 23.24 | 23.02 | 23.34 | 23.31 | 23.52 | 23.44 | 23.43 | 23.13 | 23.19 |
| ***ilvC*** | 17.43 | 16.10 | 20.40 | 20.54 | 18.55 | 18.38 | 18.23 | 18.49 | 19.91 | 19.48 | 18.85 | 18.76 |
| ***ilvE1*** | 15.81 | 15.51 | 18.18 | 17.81 | 16.38 | 15.66 | 16.20 | 16.11 | 16.99 | 16.95 | 16.01 | 16.05 |
| ***ilvE2*** | 17.05 | 17.08 | 19.05 | 19.29 | 17.17 | 17.04 | 17.53 | 17.37 | 18.44 | 18.80 | 17.58 | 17.49 |
| ***ilvF1*** | 18.12 | 18.27 | 19.71 | 19.55 | 18.81 | 18.60 | 19.00 | 18.97 | 19.71 | 19.68 | 19.33 | 19.18 |
| ***ilvF6*** | 18.88 | 19.02 | 19.07 | 18.95 | 19.52 | 18.81 | 19.37 | 19.29 | 19.78 | 18.58 | 19.63 | 19.50 |
| ***leuA1*** | 18.07 | 23.40 | 20.31 | 20.04 | 18.35 | 18.29 | 19.14 | 18.88 | 19.96 | 19.64 | 18.40 | 18.29 |
| ***leuA2*** | 15.50 | 15.39 | 17.70 | 17.80 | 16.31 | 16.48 | 16.45 | 16.33 | 17.41 | 17.31 | 17.07 | 16.83 |
| ***leuB1*** | 17.10 | 17.35 | 17.73 | 17.69 | 17.71 | 17.52 | 17.44 | 17.70 | 18.05 | 17.78 | 17.44 | 17.55 |
| ***leuB2*** | 17.19 | 17.17 | 17.68 | 17.80 | 17.70 | 17.46 | 17.67 | 17.59 | 18.10 | 18.08 | 17.41 | 17.59 |
| ***batA*** | 16.71 | 16.67 | 19.75 | 19.73 | 17.90 | 17.75 | 17.66 | 17.36 | 18.53 | 18.46 | 17.48 | 17.63 |
| ***batC*** | 17.03 | 17.10 | 20.03 | 19.84 | 17.80 | 18.03 | 18.25 | 18.29 | 18.88 | 19.14 | 18.34 | 18.25 |

| gene | AMM + Leu 5d | | | | | | AMM + Leu 7d | | | | | |
|---|---|---|---|---|---|---|---|---|---|---|---|---|
| ***gpdA*** | 14.04 | 15.19 | 15.96 | 13.89 | 15.34 | 15.76 | 15.35 | 14.61 | 15.53 | 15.14 | 14.69 | 15.22 |
| ***actB*** | 17.07 | 18.96 | 20.07 | 17.01 | 18.92 | 19.65 | 18.02 | 17.75 | 18.37 | 18.06 | 17.76 | 18.26 |
| ***malA*** | 19.42 | 18.40 | 21.54 | 19.54 | 18.33 | 21.59 | 17.60 | 17.18 | 17.17 | 17.45 | 17.25 | 17.27 |
| ***ilvA*** | 21.07 | 22.47 | 23.28 | 20.85 | 22.95 | 23.06 | 23.07 | 21.64 | 22.30 | 23.12 | 21.59 | 22.26 |
| ***ilvB*** | 25.01 | 25.71 | 25.77 | 24.89 | 26.04 | 25.58 | 26.80 | 25.39 | 25.07 | 27.00 | 25.25 | 25.42 |
| ***ilvC*** | 19.09 | 19.78 | 21.22 | 18.41 | 20.07 | 21.17 | 20.33 | 19.31 | 19.96 | 19.92 | 19.20 | 19.98 |
| ***ilvE1*** | 16.40 | 18.82 | 19.14 | 16.51 | 17.73 | 19.26 | 19.25 | 17.18 | 18.00 | 19.52 | 17.11 | 18.12 |

| | | | | | | | | | | | | |
|---|---|---|---|---|---|---|---|---|---|---|---|---|
| ***ilvE2*** | 17.20 | 19.09 | 19.26 | 16.87 | 18.93 | 19.46 | 18.87 | 17.84 | 18.38 | 19.16 | 17.67 | 18.58 |
| ***ilvF1*** | 19.69 | 21.26 | 21.29 | 19.71 | 20.88 | 21.27 | 21.49 | 20.87 | 21.16 | 21.39 | 20.51 | 21.13 |
| ***ilvF6*** | 20.06 | 21.07 | 22.15 | 20.07 | 21.44 | 22.06 | 21.65 | 21.32 | 21.51 | 20.80 | 21.19 | 21.85 |
| ***leuA1*** | 18.66 | 20.86 | 21.95 | 18.60 | 20.87 | 21.75 | 20.37 | 19.71 | 20.76 | 20.53 | 19.90 | 20.58 |
| ***leuA2*** | 17.24 | 18.12 | 18.33 | 17.22 | 18.03 | 18.34 | 17.97 | 17.77 | 18.08 | 18.29 | 17.76 | 18.06 |
| ***leuB1*** | 17.03 | 18.51 | 18.99 | 17.11 | 18.54 | 18.97 | 19.28 | 17.97 | 18.55 | 19.52 | 18.08 | 18.53 |
| ***leuB2*** | 17.62 | 19.27 | 19.49 | 17.88 | 19.12 | 19.57 | 19.56 | 18.14 | 19.09 | 19.35 | 18.22 | 19.04 |
| ***batA*** | 17.56 | 18.84 | 19.39 | 17.54 | 18.81 | 19.31 | 18.97 | 17.98 | 18.62 | 18.73 | 18.04 | 18.56 |
| ***batC*** | 18.21 | 18.98 | 19.52 | 18.08 | 18.99 | 19.33 | 19.60 | 18.45 | 19.00 | 19.71 | 18.20 | 18.72 |

| **gene** | **AMM + Val 5d** | | | | | | **AMM + Val 7d** | | | | | |
|---|---|---|---|---|---|---|---|---|---|---|---|---|
| ***gpdA*** | 14.48 | 14.25 | 15.29 | 14.11 | 14.66 | 15.03 | 16.20 | 14.29 | 14.45 | 15.80 | 14.22 | 14.08 |
| ***actB*** | 17.32 | 17.07 | 17.71 | 17.38 | 17.17 | 17.33 | 18.29 | 17.84 | 17.30 | 18.35 | 17.76 | 17.47 |
| ***malA*** | 21.64 | 19.19 | 19.84 | 21.27 | 19.35 | 19.80 | 19.62 | 21.13 | 19.53 | 19.95 | 21.13 | 19.63 |
| ***ilvA*** | 20.02 | 20.02 | 20.33 | 19.99 | 20.20 | 20.26 | 21.32 | 20.82 | 20.33 | 21.32 | 20.91 | 20.33 |
| ***ilvB*** | 24.01 | 23.92 | 24.30 | 23.08 | 23.78 | 24.30 | 25.67 | 24.37 | 24.13 | 25.41 | 24.28 | 24.17 |
| ***ilvC*** | 18.89 | 17.39 | 18.26 | 18.83 | 17.92 | 18.25 | 19.35 | 19.12 | 18.14 | 19.53 | 18.94 | 18.47 |
| ***ilvE1*** | 16.41 | 16.31 | 16.13 | 16.62 | 16.04 | 16.22 | 18.36 | 16.61 | 16.57 | 17.98 | 16.34 | 16.44 |
| ***ilvE2*** | 17.55 | 16.90 | 17.36 | 18.32 | 16.89 | 17.62 | 18.94 | 16.98 | 17.12 | 19.21 | 16.87 | 17.10 |
| ***ilvF1*** | 19.14 | 19.05 | 19.20 | 19.15 | 18.98 | 19.05 | 19.98 | 19.52 | 19.29 | 20.13 | 19.41 | 19.31 |
| ***ilvF6*** | 19.17 | 20.47 | 20.47 | 19.29 | 20.64 | 20.62 | 21.01 | 21.00 | 20.49 | 21.07 | 20.97 | 20.48 |
| ***leuA1*** | 17.64 | 18.57 | 18.94 | 17.94 | 18.70 | 18.93 | 19.59 | 19.37 | 19.32 | 19.46 | 19.26 | 19.39 |
| ***leuA2*** | 16.05 | 16.55 | 16.82 | 16.00 | 16.60 | 16.81 | 17.35 | 17.38 | 16.51 | 17.30 | 17.35 | 16.56 |
| ***leuB1*** | 16.07 | 17.23 | 17.41 | 16.06 | 17.36 | 17.36 | 18.37 | 17.53 | 17.11 | 18.36 | 17.53 | 17.18 |
| ***leuB2*** | 17.14 | 17.39 | 17.42 | 17.17 | 17.35 | 17.65 | 18.63 | 17.37 | 17.50 | 18.89 | 17.53 | 17.39 |
| ***batA*** | 18.26 | 17.60 | 17.96 | 18.24 | 17.56 | 17.94 | 18.86 | 17.86 | 17.80 | 19.24 | 17.93 | 17.65 |
| ***batC*** | 17.50 | 17.34 | 17.87 | 17.50 | 17.56 | 17.72 | 18.39 | 18.27 | 17.93 | 18.37 | 18.21 | 18.00 |

| **gene** | **AMM + Ile/Leu/Val** | | | | | | **AMM + Ile/Leu/Val** | | | | | |
|---|---|---|---|---|---|---|---|---|---|---|---|---|
| ***gpdA*** | 14.44 | 13.77 | 14.27 | 14.15 | 13.19 | 14.00 | 14.24 | 14.63 | 15.04 | 14.01 | 14.68 | 14.30 |
| ***actB*** | 17.24 | 16.25 | 16.48 | 17.03 | 16.33 | 16.35 | 17.11 | 17.09 | 16.94 | 16.92 | 17.18 | 16.74 |
| ***malA*** | 21.11 | 20.96 | 20.49 | 21.13 | 20.70 | 20.54 | 22.08 | 20.19 | 21.50 | 21.23 | 20.26 | 21.44 |
| ***ilvA*** | 21.60 | 21.35 | 21.48 | 21.53 | 21.26 | 21.37 | 22.09 | 22.97 | 21.65 | 22.19 | 22.62 | 21.90 |
| ***ilvB*** | 24.12 | 23.43 | 24.89 | 24.28 | 24.00 | 23.60 | 26.84 | 23.02 | 23.45 | 26.48 | 23.63 | 23.76 |
| ***ilvC*** | 19.22 | 18.73 | 19.14 | 19.17 | 18.91 | 18.89 | 19.55 | 20.09 | 20.06 | 19.40 | 20.21 | 20.14 |
| ***ilvE1*** | 17.69 | 17.17 | 17.18 | 17.52 | 17.08 | 17.04 | 18.06 | 19.42 | 18.04 | 18.08 | 19.42 | 18.04 |
| ***ilvE2*** | 17.05 | 16.40 | 17.00 | 17.28 | 16.64 | 17.24 | 16.78 | 17.96 | 17.06 | 17.08 | 17.95 | 17.40 |
| ***ilvF1*** | 21.30 | 21.08 | 20.91 | 21.22 | 21.19 | 21.22 | 16.12 | 18.13 | 19.71 | 13.90 | 17.90 | 19.77 |
| ***ilvF6*** | 21.00 | 21.03 | 20.34 | 21.14 | 21.10 | 20.44 | 21.36 | 20.71 | 21.54 | 22.70 | 20.70 | 21.94 |
| ***leuA1*** | 19.77 | 19.36 | 19.28 | 19.71 | 19.45 | 19.20 | 20.66 | 20.44 | 20.52 | 20.42 | 20.46 | 20.51 |
| ***leuA2*** | 18.46 | 17.99 | 18.08 | 18.53 | 17.93 | 18.02 | 18.70 | 18.85 | 17.86 | 18.97 | 18.50 | 17.57 |
| ***leuB1*** | 18.16 | 17.58 | 17.75 | 18.23 | 17.69 | 17.86 | 18.25 | 19.04 | 17.43 | 18.20 | 19.12 | 17.86 |
| ***leuB2*** | 18.19 | 17.96 | 17.97 | 18.32 | 18.01 | 17.67 | 18.27 | 18.91 | 18.66 | 18.29 | 18.87 | 18.23 |
| ***batA*** | 17.38 | 16.85 | 17.07 | 17.47 | 17.06 | 17.21 | 17.36 | 17.95 | 16.92 | 17.35 | 17.86 | 17.80 |
| ***batC*** | 18.83 | 18.17 | 18.62 | 18.78 | 18.25 | 18.35 | 18.87 | 19.95 | 18.29 | 18.79 | 19.71 | 18.89 |

**Table S7. Statistical analysis of gene expression data according to One-Way-ANOVA tests.** Significance values are indicated as: ns, not significant; * p<0.05, ** p<0.01; *** p<0.001 and **** p<0.0001.

| | Mean Diff. | 95.00% CI of diff. | Summary | P Value |
|---|---|---|---|---|
| ***malA*** | | | | |
| **5d vs. 7d** | -0.258 | -1.301 to 0.7846 | ns | 0.9916 |
| **5d vs. 5d** | -1.316 | -2.359 to -0.2739 | ** | 0.0057 |
| **5d vs. 7d** | -0.8179 | -1.861 to 0.2246 | ns | 0.1998 |
| **5d vs. 5d** | 1.899 | 0.8568 to 2.942 | **** | <0.0001 |
| **5d vs. 7d** | 1.532 | 0.4897 to 2.575 | *** | 0.0007 |
| **5d vs. 5d** | 0.7001 | -0.3425 to 1.743 | ns | 0.3557 |
| **5d vs. 7d** | -1.417 | -2.46 to -0.3744 | ** | 0.0022 |
| **5d vs. 5d** | 3.204 | 2.162 to 4.247 | **** | <0.0001 |
| **5d vs. 7d** | 3.034 | 1.991 to 4.076 | **** | <0.0001 |
| ***ilvA*** | | | | |
| **5d vs. 7d** | -0.3107 | -1.353 to 0.7319 | ns | 0.9731 |
| **5d vs. 5d** | -1.828 | -2.871 to -0.7859 | **** | <0.0001 |
| **5d vs. 7d** | -2.087 | -3.13 to -1.045 | **** | <0.0001 |
| **5d vs. 5d** | -0.6658 | -1.708 to 0.3767 | ns | 0.4124 |
| **5d vs. 7d** | -0.3605 | -1.403 to 0.6821 | ns | 0.9368 |
| **5d vs. 5d** | -1.893 | -2.936 to -0.8505 | **** | <0.0001 |
| **5d vs. 7d** | -1.571 | -2.614 to -0.5288 | *** | 0.0005 |
| **5d vs. 5d** | 0.04481 | -0.9978 to 1.087 | ns | 0.9999 |
| **5d vs. 7d** | 0.3719 | -0.6707 to 1.414 | ns | 0.9254 |
| ***ilvB*** | | | | |
| **5d vs. 7d** | -0.3166 | -1.359 to 0.726 | ns | 0.9698 |
| **5d vs. 5d** | -1.664 | -2.707 to -0.6215 | *** | 0.0002 |
| **5d vs. 7d** | -1.605 | -2.648 to -0.5627 | *** | 0.0003 |
| **5d vs. 5d** | 0.3644 | -0.6782 to 1.407 | ns | 0.933 |
| **5d vs. 7d** | 0.9477 | -0.09492 to 1.99 | ns | 0.0938 |
| **5d vs. 5d** | -0.4361 | -1.479 to 0.6065 | ns | 0.841 |
| **5d vs. 7d** | 0.001112 | -1.041 to 1.044 | ns | >0.9999 |
| **5d vs. 5d** | 0.416 | -0.6266 to 1.459 | ns | 0.8709 |
| **5d vs. 7d** | 0.4522 | -0.5904 to 1.495 | ns | 0.8149 |
| ***ilvC*** | | | | |
| **5d vs. 7d** | 0.0578 | -0.9848 to 1.1 | ns | 0.9998 |
| **5d vs. 5d** | -0.98 | -2.023 to 0.0626 | ns | 0.0763 |
| **5d vs. 7d** | -0.6049 | -1.647 to 0.4377 | ns | 0.5237 |
| **5d vs. 5d** | 0.1273 | -0.9153 to 1.17 | ns | 0.9995 |
| **5d vs. 7d** | 0.2165 | -0.8261 to 1.259 | ns | 0.9966 |
| **5d vs. 5d** | -0.6856 | -1.728 to 0.3569 | ns | 0.3792 |
| **5d vs. 7d** | -0.3907 | -1.433 to 0.6519 | ns | 0.9042 |
| **5d vs. 5d** | 0.7381 | -0.3044 to 1.781 | ns | 0.2986 |
| **5d vs. 7d** | 1.161 | 0.1189 to 2.204 | * | 0.0207 |

| | | | | |
|---|---|---|---|---|
| ***ilvE1*** | | | | |
| **5d vs. 7d** | 0.4549 | -0.5876 to 1.498 | ns | 0.8101 |
| **5d vs. 5d** | -2.06 | -3.103 to -1.017 | **** | <0.0001 |
| **5d vs. 7d** | -2.231 | -3.274 to -1.189 | **** | <0.0001 |
| **5d vs. 5d** | -0.8955 | -1.938 to 0.1471 | ns | 0.1291 |
| **5d vs. 7d** | -0.4194 | -1.462 to 0.6231 | ns | 0.8659 |
| **5d vs. 5d** | -1.734 | -2.777 to -0.6919 | **** | <0.0001 |
| **5d vs. 7d** | -1.329 | -2.372 to -0.2864 | ** | 0.0051 |
| **5d vs. 5d** | -0.05648 | -1.099 to 0.9861 | ns | 0.9998 |
| **5d vs. 7d** | 0.716 | -0.3266 to 1.759 | ns | 0.3311 |
| ***ilvE2*** | | | | |
| **5d vs. 7d** | 0.2383 | -0.8043 to 1.281 | ns | 0.9935 |
| **5d vs. 5d** | -0.497 | -1.54 to 0.5455 | ns | 0.7341 |
| **5d vs. 7d** | -0.4108 | -1.453 to 0.6318 | ns | 0.8782 |
| **5d vs. 5d** | -0.06724 | -1.11 to 0.9753 | ns | 0.9997 |
| **5d vs. 7d** | 0.1393 | -0.9033 to 1.182 | ns | 0.9995 |
| **5d vs. 5d** | -0.2342 | -1.277 to 0.8084 | ns | 0.994 |
| **5d vs. 7d** | -0.3299 | -1.373 to 0.7126 | ns | 0.9615 |
| **5d vs. 5d** | -0.03858 | -1.081 to 1.004 | ns | 0.9999 |
| **5d vs. 7d** | -0.05913 | -1.102 to 0.9835 | ns | 0.9998 |
| ***ilvF1*** | | | | |
| **5d vs. 7d** | -0.2281 | -1.271 to 0.8145 | ns | 0.9963 |
| **5d vs. 5d** | -2.126 | -3.168 to -1.083 | **** | <0.0001 |
| **5d vs. 7d** | -1.674 | -2.717 to -0.6315 | *** | 0.0002 |
| **5d vs. 5d** | -0.5957 | -1.638 to 0.4469 | ns | 0.5413 |
| **5d vs. 7d** | 0.05393 | -0.9886 to 1.097 | ns | 0.9998 |
| **5d vs. 5d** | -1.29 | -2.332 to -0.2471 | ** | 0.0072 |
| **5d vs. 7d** | -1.149 | -2.192 to -0.1067 | * | 0.0228 |
| **5d vs. 5d** | 1.384 | 0.341 to 2.426 | ** | 0.0031 |
| **5d vs. 7d** | -1.256 | -2.422 to -0.09061 | * | 0.0277 |
| ***ilvF2*** | | | | |
| **5d vs. 7d** | -0.5958 | -1.638 to 0.4468 | ns | 0.541 |
| **5d vs. 5d** | -2.129 | -3.171 to -1.086 | **** | <0.0001 |
| **5d vs. 7d** | -1.829 | -2.871 to -0.7861 | **** | <0.0001 |
| **5d vs. 5d** | -0.379 | -1.422 to 0.6636 | ns | 0.9178 |
| **5d vs. 7d** | 0.1122 | -0.9304 to 1.155 | ns | 0.9996 |
| **5d vs. 5d** | -0.5194 | -1.562 to 0.5232 | ns | 0.6911 |
| **5d vs. 7d** | -0.1846 | -1.227 to 0.8579 | ns | 0.9979 |
| **5d vs. 5d** | 0.8662 | -0.1764 to 1.909 | ns | 0.153 |
| **5d vs. 7d** | 1.032 | -0.0109 to 2.074 | ns | 0.0539 |
| ***leuA1*** | | | | |
| **5d vs. 7d** | -0.3108 | -1.353 to 0.7318 | ns | 0.973 |
| **5d vs. 5d** | 0.02203 | -1.021 to 1.065 | ns | >0.9999 |

| | | | | |
|---|---|---|---|---|
| **5d vs. 7d** | -0.6366 | -1.679 to 0.406 | ns | 0.4644 |
| **5d vs. 5d** | 0.4566 | -0.586 to 1.499 | ns | 0.8073 |
| **5d vs. 7d** | 0.5774 | -0.4652 to 1.62 | ns | 0.5769 |
| **5d vs. 5d** | -0.6159 | -1.658 to 0.4267 | ns | 0.5027 |
| **5d vs. 7d** | -0.06528 | -1.108 to 0.9773 | ns | 0.9998 |
| **5d vs. 5d** | 1.04 | -0.002379 to 2.083 | ns | 0.0509 |
| **5d vs. 7d** | 1.586 | 0.5438 to 2.629 | *** | 0.0004 |
| | | | | |
| ***leuA2*** | | | | |
| **5d vs. 7d** | -0.07575 | -1.118 to 0.9668 | ns | 0.9997 |
| **5d vs. 5d** | -1.18 | -2.223 to -0.1376 | * | 0.0179 |
| **5d vs. 7d** | -0.8249 | -1.867 to 0.2177 | ns | 0.1924 |
| **5d vs. 5d** | -0.1315 | -1.174 to 0.9111 | ns | 0.9995 |
| **5d vs. 7d** | 0.2293 | -0.8133 to 1.272 | ns | 0.9963 |
| **5d vs. 5d** | -0.6408 | -1.683 to 0.4018 | ns | 0.4566 |
| **5d vs. 7d** | -0.4156 | -1.458 to 0.6269 | ns | 0.8714 |
| **5d vs. 5d** | 1.674 | 0.6315 to 2.717 | *** | 0.0002 |
| **5d vs. 7d** | 1.455 | 0.4125 to 2.498 | ** | 0.0015 |
| | | | | |
| ***leuB1*** | | | | |
| **5d vs. 7d** | -0.141 | -1.184 to 0.9016 | ns | 0.9994 |
| **5d vs. 5d** | -1.088 | -2.13 to -0.0451 | * | 0.0362 |
| **5d vs. 7d** | -0.953 | -1.996 to 0.08963 | ns | 0.0908 |
| **5d vs. 5d** | -0.694 | -1.737 to 0.3486 | ns | 0.3655 |
| **5d vs. 7d** | -0.0009555 | -1.044 to 1.042 | ns | >0.9999 |
| **5d vs. 5d** | -1.058 | -2.101 to -0.01564 | * | 0.0448 |
| **5d vs. 7d** | -0.6899 | -1.732 to 0.3527 | ns | 0.3721 |
| **5d vs. 5d** | 0.5375 | -0.5051 to 1.58 | ns | 0.6557 |
| **5d vs. 7d** | 0.5035 | -0.5391 to 1.546 | ns | 0.7219 |
| | | | | |
| ***leuB2*** | | | | |
| **5d vs. 7d** | -0.0686 | -1.111 to 0.974 | ns | 0.9997 |
| **5d vs. 5d** | -1.222 | -2.265 to -0.1796 | * | 0.0127 |
| **5d vs. 7d** | -0.9943 | -2.037 to 0.04833 | ns | 0.0695 |
| **5d vs. 5d** | -0.2109 | -1.254 to 0.8317 | ns | 0.9968 |
| **5d vs. 7d** | 0.1194 | -0.9231 to 1.162 | ns | 0.9996 |
| **5d vs. 5d** | -0.7678 | -1.81 to 0.2748 | ns | 0.2586 |
| **5d vs. 7d** | -0.6168 | -1.659 to 0.4258 | ns | 0.501 |
| **5d vs. 5d** | 0.5572 | -0.4854 to 1.6 | ns | 0.6169 |
| **5d vs. 7d** | 0.5996 | -0.443 to 1.642 | ns | 0.5337 |
| | | | | |
| ***batA*** | | | | |
| **5d vs. 7d** | -0.3043 | -1.347 to 0.7383 | ns | 0.9764 |
| **5d vs. 5d** | -1.32 | -2.362 to -0.2773 | ** | 0.0055 |
| **5d vs. 7d** | -1.541 | -2.584 to -0.4988 | *** | 0.0007 |
| **5d vs. 5d** | -0.8744 | -1.917 to 0.1682 | ns | 0.1461 |
| **5d vs. 7d** | -0.9486 | -1.991 to 0.09397 | ns | 0.0933 |
| **5d vs. 5d** | -1.096 | -2.139 to -0.05382 | * | 0.034 |

| | | | | |
|---|---|---|---|---|
| **5d vs. 7d** | -0.9271 | -1.97 to 0.1155 | ns | 0.1066 |
| **5d vs. 5d** | -0.8974 | -1.94 to 0.1452 | ns | 0.1276 |
| **5d vs. 7d** | -1 | -2.043 to 0.04263 | ns | 0.0669 |
| | | | | |
| ***batC*** | | | | |
| **5d vs. 7d** | 0.1448 | -0.8977 to 1.187 | ns | 0.9994 |
| **5d vs. 5d** | -1.06 | -2.103 to -0.01738 | * | 0.0442 |
| **5d vs. 7d** | -0.8489 | -1.892 to 0.1936 | ns | 0.1686 |
| **5d vs. 5d** | -1.165 | -2.208 to -0.1229 | * | 0.0201 |
| **5d vs. 7d** | -0.9252 | -1.968 to 0.1174 | ns | 0.108 |
| **5d vs. 5d** | -0.7768 | -1.819 to 0.2658 | ns | 0.2472 |
| **5d vs. 7d** | -0.429 | -1.472 to 0.6136 | ns | 0.8519 |
| **5d vs. 5d** | 0.4131 | -0.6295 to 1.456 | ns | 0.875 |
| **5d vs. 7d** | 0.5249 | -0.5177 to 1.567 | ns | 0.6804 |

**Table S8. Amino acid sequences used for phylogenetic analysis of BCAA aminotransferase homologs.**

| Organism | Protein | Accession number | reference |
|---|---|---|---|
| *Aspergillus nidulans* | BatA | CBF77764.1 | [16] |
| *Arabidopsis thaliana* | BCAT1 | BAH19488.1 | [17] |
| *Arabidopsis thaliana* | BCAT2L | AEE28538.1 | [17] |
| *Arabidopsis thaliana* | BCAT2S | AEE28539.1 | [17] |
| *Arabidopsis thaliana* | BCAT3 | Q9M401.1 | [17] |
| *Arabidopsis thaliana* | BCAT4 | Q9LE06.1 | [17] |
| *Arabidopsis thaliana* | BCAT5 | Q9FYA6.1 | [17] |
| *Bacillus subtilis* | IlvE | sp\|O31461 | [18] |
| *Bacillus subtilis* | IlvE2/IlvK | sp\|P39576 | |
| *Cryptococcus neoformans* | BatA | XP_771917.1 | [19] |
| *Cryptococcus neoformans var. grubii* | BatA | OXB33852.1 | [19] |
| *Chlamydomonas reinhardtii* | BCA1 | Cre02.g081400.t1.2 | [20] |
| *Chlamydomonas reinhardtii* | BCA2 | Cre05.g245900.t1.2 | [20] |
| *Chlamydomonas reinhardtii* | BCA3 | Cre10.g458050.t1.2 | [20] |
| *Chlamydomonas reinhardtii* | BCA4 | Cre13.g576400.t1.2 | [20] |
| *Escherichia coli* | IlvE | sp\|P0AB80 | [21] |
| *Fusarium graminearum* | BatA | XP_011328206.1 | |
| *Glycine max* | BCAT1 | Glyma.06G050100.1 | [22] |
| *Glycine max* | BCAT2 | Glyma.04G049200.1 | |
| *Glycine max* | BCAT3 | Glyma.08G063000.1 | |
| *Haplosporangium gracile* | BatA | KAF8942323.1 | |
| *human* | mBCAT1 | sp\|P54687 | [23] |
| *Linnemannia elongata* | BatA | KAF9288437.1 | |
| *Methanococcus aeolicus* | IlvE | WP_011973904.1 | |
| *Mortierella alpina* | BatA | **ADAG01001070** | **This study.** |
| *Mortierella alpina* | BatC | **ADAG01001070** | **This study**. |
| *Mycobacterium tuberculosis* | Bat1 | PLV46681.1 | |
| *Mycobacterium tuberculosis* | Bat2 | CNG44365.1 | |
| *Pseudomonas aeruginosa* | IlvE | sp\|O86428\| | |
| *Phycomyces blakesleeanus* | BatA | XP_018292219.1 | |
| *Pyricularia* (syn. *Magnaporthe*) *oryzae* | BAT1 | XP_003709286.1 | [13] |
| *Pyricularia* (syn. *Magnaporthe*) *oryzae* | BAT3 | XP_003708897.1 | [13] |
| *Physcomitrella patens* | IlvE2 | Pp3c3_15010V3.1 | |
| *Physcomitrella patens* | IlvE3 | Pp3c10_12890V3.1 | |
| *Populus trichocarpa* | BCAT2 | Potri.002G113600.1 | |
| *Populus trichocarpa* | BCAT4 | Potri.009G082600.1 | |
| *Podila verticillata* | BatA | KAF9384042.1 | |
| *Streptomyces albus* | Bat1 | WP_032780549.1 | |
| *Saccharomyces cerevisiae* | BAT1 | NP_012078.3 | [24] |
| *Saccharomyces cerevisiae* | BAT2 | NP_012682.1 | [24] |
| *Solanum lycopersicum* | BCAT1 | XP_010314598.1 | [25] |
| *Solanum lycopersicum* | BCAT2 | XP_010323427.1 | [25] |
| *Solanum lycopersicum* | BCAT3 | XP_004231952.1 | [25] |
| *Solanum lycopersicum* | BCAT4 | XP_004234645.1 | [25] |
| *Solanum lycopersicum* | BCAT5 | XP_004253486.3 | [25] |
| *Solanum lycopersicum* | BCAT6 | XP_019069001.1 | [25] |
| *Trifolium pratense* | BCAT1 | Tp57577_TGAC_v2_mRNA23101 | |
| *Trifolium pratense* | BCAT2 | Tp57577_TGAC_v2_mRNA2817 | |
| *Trifolium pratense* | BCAT3 | Tp57577_TGAC_v2_mRNA31240 | |
| *Trifolium pratense* | BCAT4 | Tp57577_TGAC_v2_mRNA3185 | |
| *Volvox carteri* | BCAT | Vocar.0026s0064.1 | |

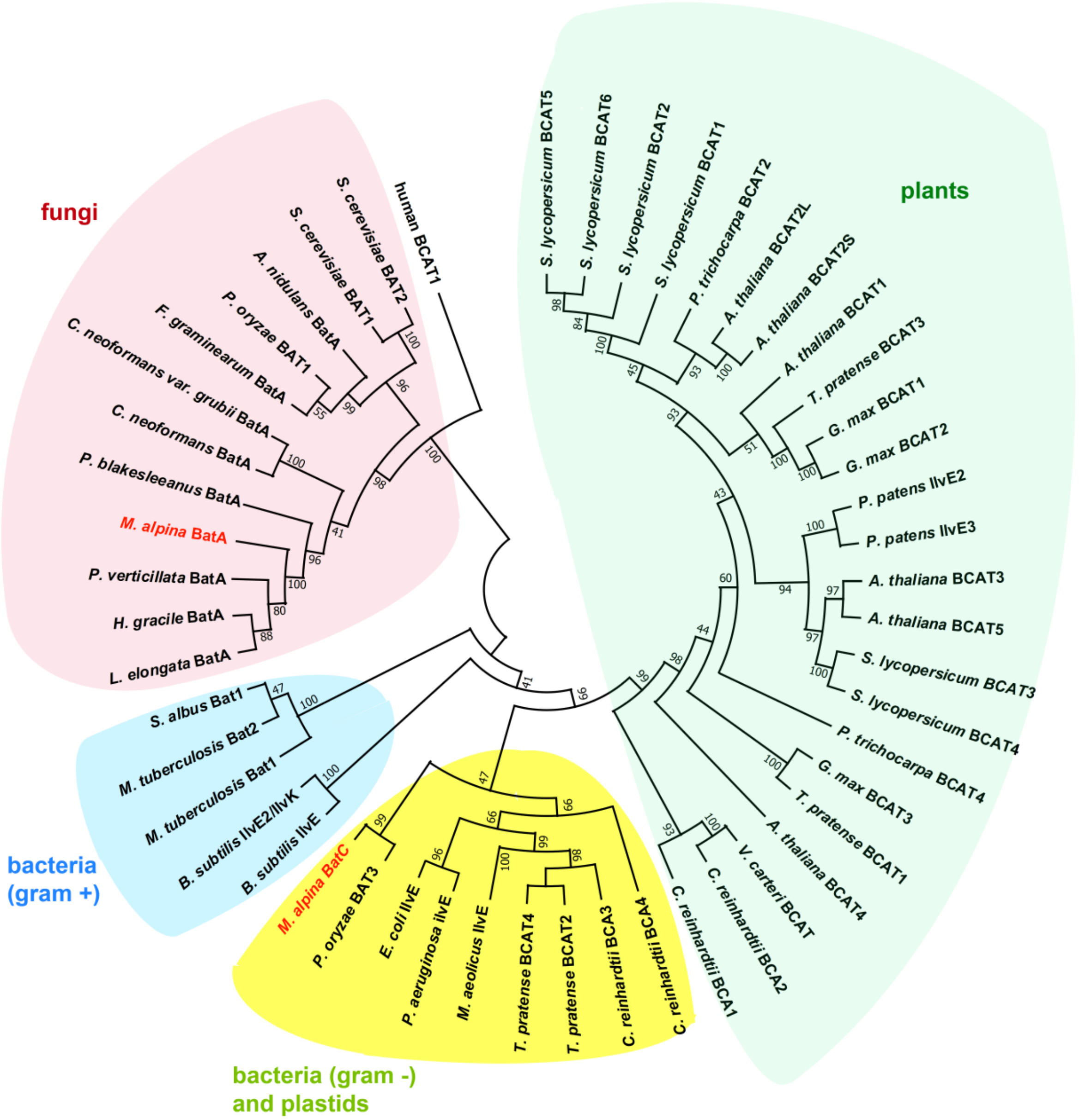


**Figure S1. Phylogenetic analysis of BCAA aminotransferases (BATs).** The evolutionary history was inferred by using the Maximum Likelihood method and Le_Gascuel_2008 model [1]. The bootstrap consensus tree inferred from 1000 replicates [2]. Initial tree(s) for the heuristic search were obtained automatically by applying Neighbor-Join and BioNJ algorithms to a matrix of pairwise distances estimated using the JTT model, and then selecting the topology with superior log likelihood value. A discrete Gamma distribution was used to model evolutionary rate differences among sites (5 categories (+G, parameter = 2.7340)). The rate variation model allowed for some sites to be evolutionarily invariable ([+I], 0.91% sites). Evolutionary analyses were conducted in MEGA11 [3]. This analysis involved 51 amino acid sequences (Table S8). The human BAT served as outgroup.

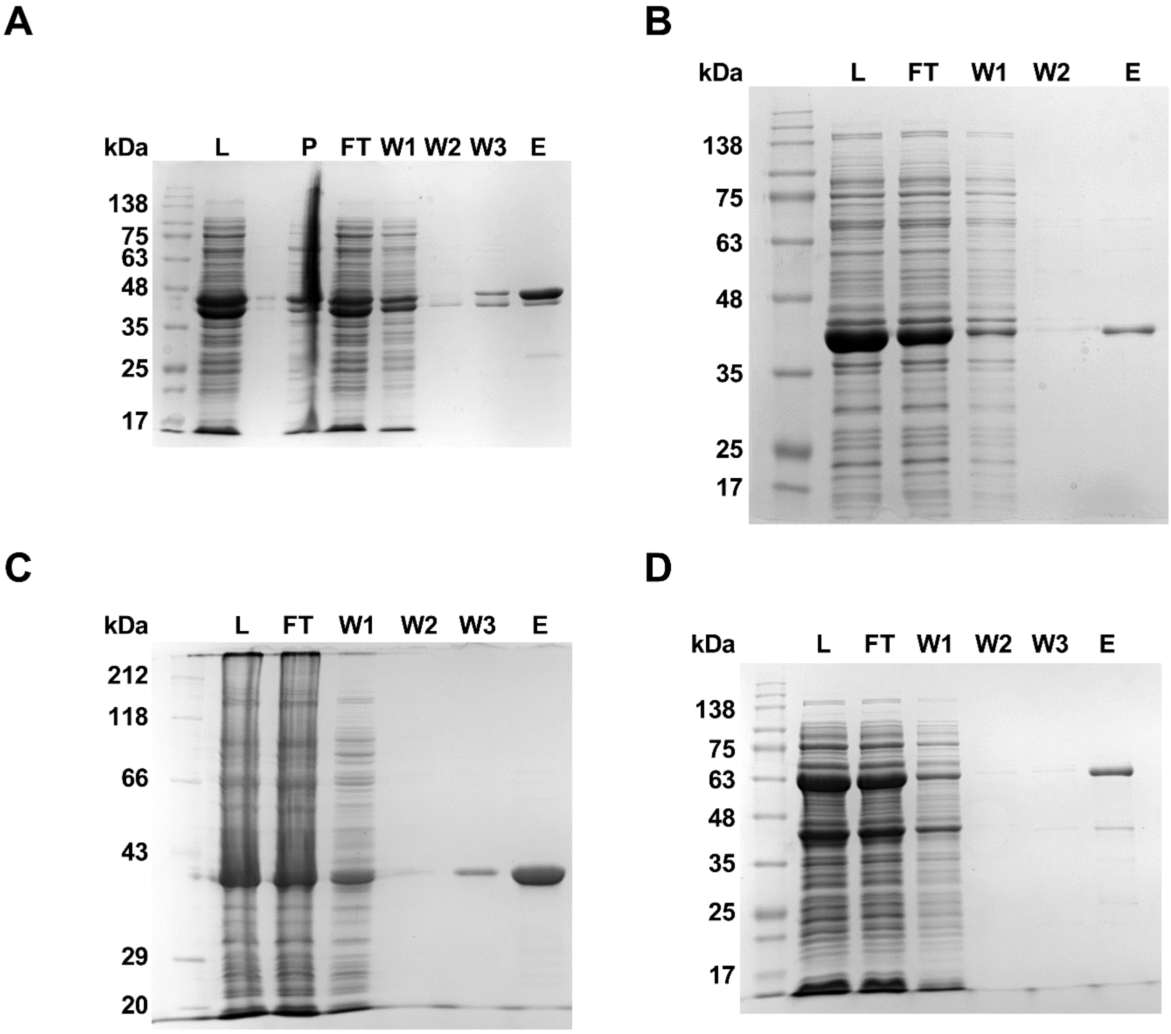


**Figure S2. SDS polyacrylamide gel electrophoresis (SDS-PAGE) of His$_6$-tagged *Mortierella alpina* enzymes.** (**A**) BatA-L (calculated protein mass 45.2 kDa), (**B**) BatA-S (41.6 kDa), (**C**) BatC (39.9 kDa) and (**D**) LeuA1 (60.1 kDa) are shown. The N-terminal His$_6$-tag adds approximately 2.0 kDa. Abbreviations: L, *E. coli* cell lysate; P, *E. coli* cell pellet, FT, flowthrough after Ni$^{2+}$-NTA binding; W1-3, wash steps with 10, 25, and 50 mM imidazole; E, elution with 250 mM imidazole. Note, that BatA-L was instable, resulting in main protein band at 45 kDa and two minor degraded proteins at ~40 kDa and ~28 kDa.

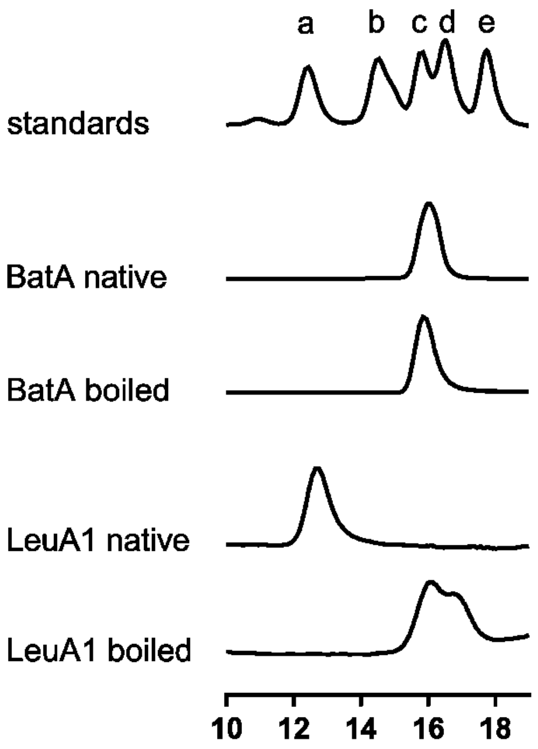


**Figure S3. Size exclusion chromatography (SEC) of His$_6$-tagged proteins**. BatA-S and LeuA1 were chromatographed on a Superdex 200 increase 10/300 GL column using an isocratic gradient. (SEC was performed with native and heat-denatured (95°C, 5 min) proteins. Native and denatured His$_6$-tagged BatA elutes at the same retention volume of 16.3 mL corresponding to a size of 50.1 kDa (calculated mass: 43.6 kDa), suggesting a monomeric enzyme. In contrast, native His$_6$-tagged LeuA1 forms a homohexamer in solution and elutes at 12.5 mL corresponding to 399.8 kDa (calc. 375 kDa), while its denatured monomer elutes at a retention volume of 16 mL with a corresponding mass of 62.9 kDa (calc. 62.5 kDa). Proteins of the GE Healthcare low and high molecular weight standard (upper lane) served as reference (a: ferritin, 440 kDa; b: aldolase, 158 kDa; c: conalbumin 75 kDa; d: ovalbumin, 43 kDa, e: carbonic anhydrase, 13.7 kDa).

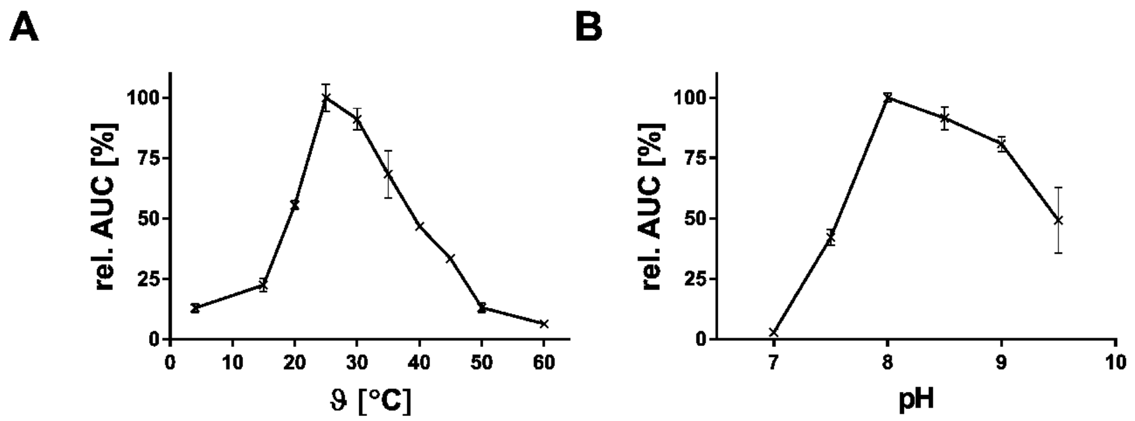


**Figure S4. Impact of temperature and pH on BatA activity**. Temperature (**A**) and pH dependence (**B**) of BatA. Error bars indicate the standard errors of the mean of three independent measurements.

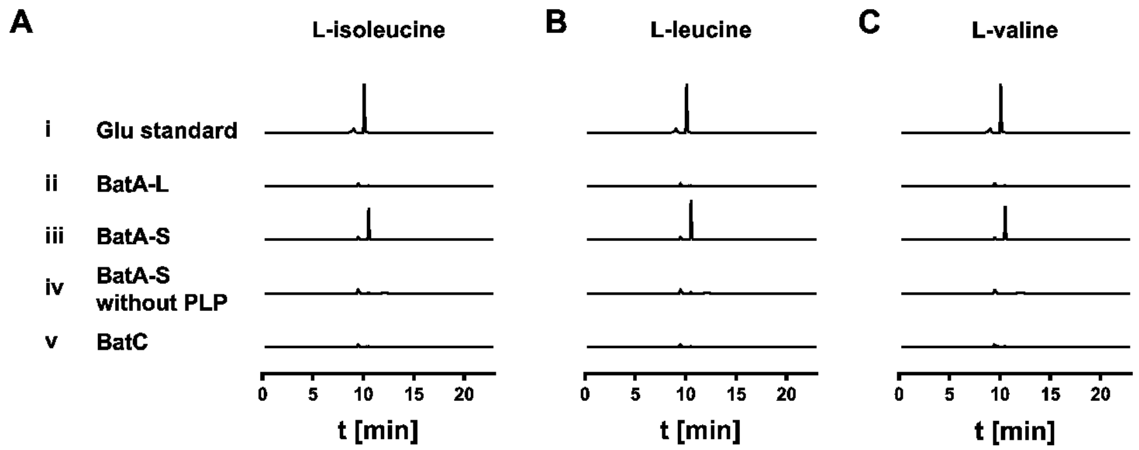


**Figure S5. Activity of Bat-L, BatA-S, and BatC.** Three different BCAAs (Ile (**A**), Leu (**B**), Val (**C**)) were used as substrates at a final concentration of 5 mM and the glutamate formation was monitored by UHPLC-MS. The extracted ion chromatogram (EIC) for the product glutamate (m/z 148.13 $[M+H]^+$) is shown in each trace. A commercial glutamate standard (trace i) served as reference. The activity was determined for BatA-L (trace ii), BatA-S (iii), Bat-S without additional pyridoxalphosphate (PLP) (iv) and BatC (v). Note that solely BatA-S converted all three BCAAs to glutamate in a PLP-dependent manner.

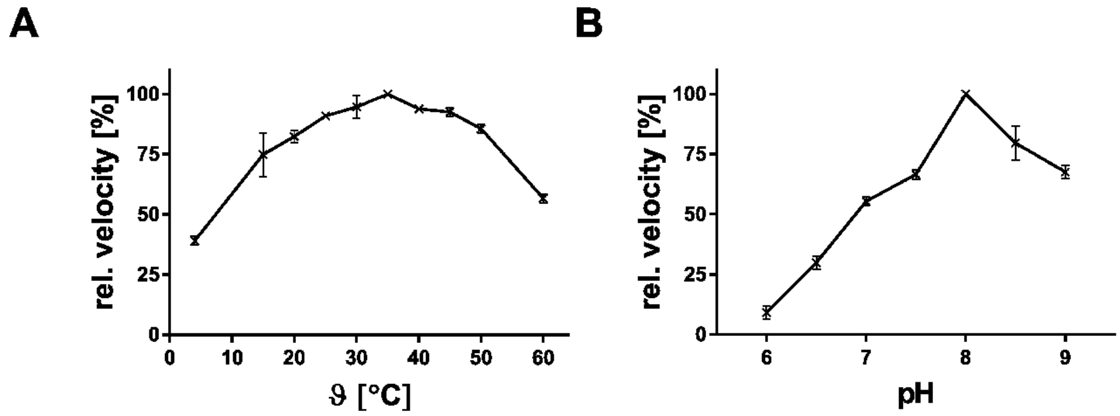


**Figure S6. Impact of temperature and pH on LeuA1 activity**. Temperature (**A**) and pH dependence (**B**) of LeuA1. Error bars indicate the standard errors of the mean of three independent measurements.

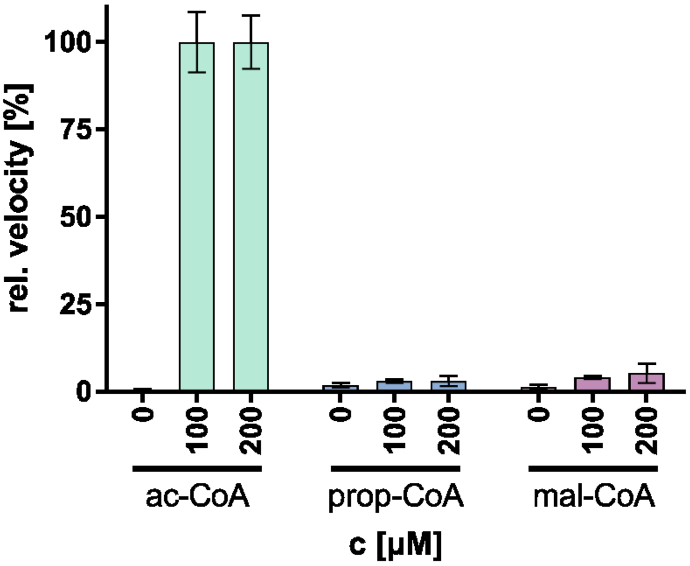


**Figure S7. Acyl-CoA dependence of LeuA1 activity**. LeuA1 was incubated with its native substrate KIV and the potential co-substrates Ac-CoA, Prop-CoA and Mal-CoA at concentrations of 0, 100, and 200 μM. Error bars indicate the standard errors of the mean of three independent measurements.

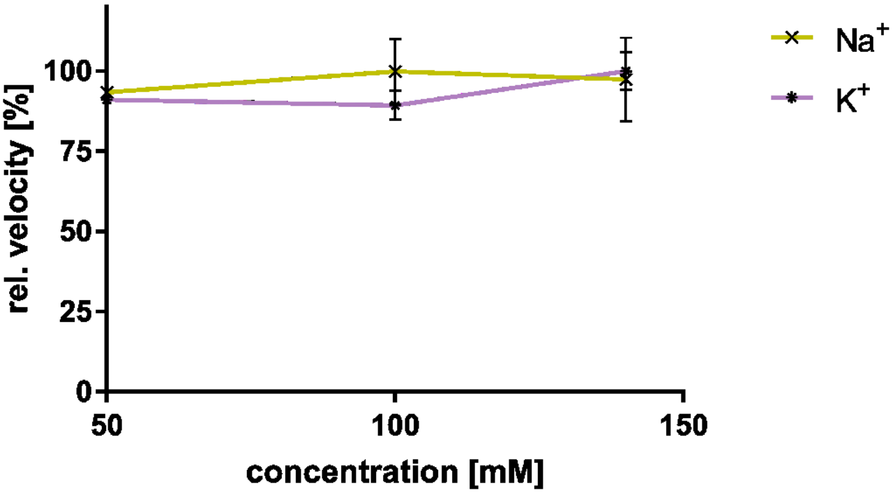


**Figure S8. Sodium and potassium dependence of LeuA1 activity**. LeuA1 was incubated with its native substrate KIV and Ac-CoA in presence of 50, 100, or 140 mM $Na^+$ or $K^+$. Enzyme activity is not affected. Error bars indicate the standard errors of the mean of three independent measurements.

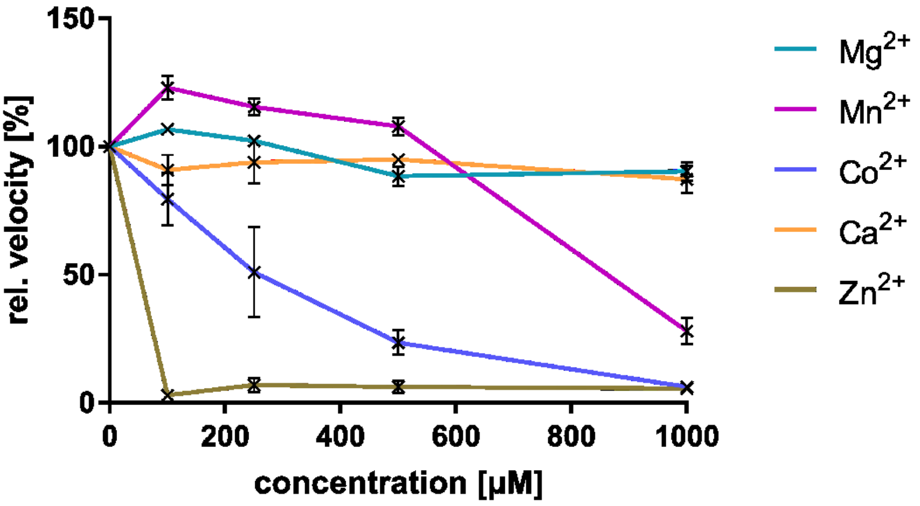


**Figure S9. Impact of divalent cations on LeuA1 activity.** LeuA1 was incubated in presence of 0, 100, 250, 500 and 1000 µM divalent ions ($Mg^{2+}$, $Ca^{2+}$, $Mn^{2+}$, $Co^{2+}$, $Zn^{2+}$). Error bars indicate the standard errors of the mean of three independent measurements.

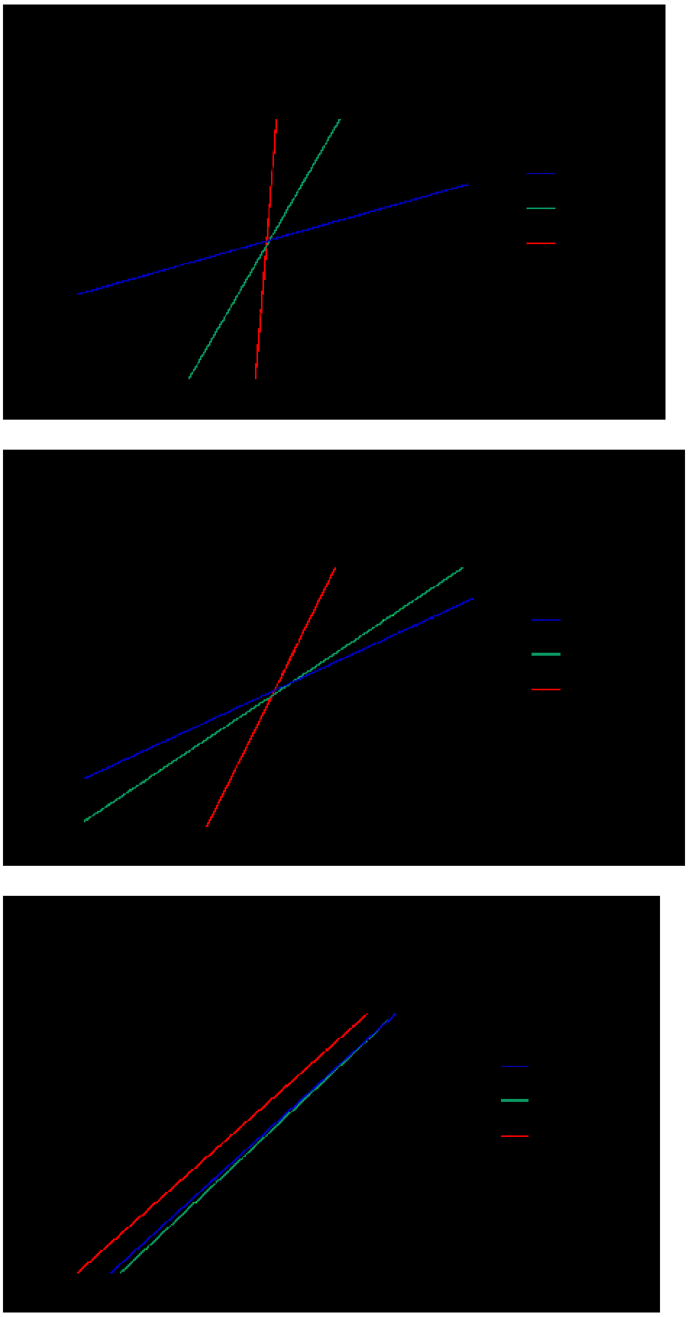

**Figure S10. Regulation of LeuA1 by primary metabolites**. Reactions were carried out using KIV and Ac-CoA as substrates. The Dixon plots are shown for reactions in the presence of (**A**) Leu (at 100 and 1000 µM), (**B**) KIC (at 100 µM and 1000 µM) and (**C**) Prop-CoA (at 10 and 100 µM). An untreated control was carried out in parallel (0 µM). Error bars indicate the standard errors of the mean of three independent measurements. Note, that Leu, KIC and prop-CoA are non-competitive ($v_{max}$ decreased, $K_M$ constant), competitive ($v_{max}$ constant, $K_M$ increased) and mixed-type inhibitors ($v_{max}$ decreased, $K_M$ decreased), respectively.

## References


1. Le, S.Q.; Gascuel, O. An improved general amino acid replacement matrix. *Mol. Biol. Evol.* **2008**, *25*, 1307-1320.
2. Felsenstein, J. Confidence limits on phylogenies: An approach using the bootstrap. *Evolution* **1985**, *39*, 783-791.
3. Tamura, K.; Stecher, G.; Kumar, S. MEGA11: Molecular evolutionary genetics analysis version 11. *Mol. Biol. Evol.* **2021**, *38*, 3022-3027.
4. de Kraker, J.W.; Luck, K.; Textor, S.; Tokuhisa, J.G.; Gershenzon, J. Two *Arabidopsis* genes (IPMS1 and IMPS2) encode isopropylmalate synthase, the branchpoint step in the biosynthesis of leucine. *Plant Physiol.* **2007**, *143*, 970-986.
5. Orasch, T.; Dietl, A.M.; Shadkchan, Y.; Binder, U.; Bauer, I.; Lass-Flörl, C.; Osherov, N.; Haas, H. The leucine biosynthetic pathway is crucial for adaptation to iron starvation and virulence in *Aspergillus fumigatus*. *Virulence* **2019**, *10*, 925-934.
6. Bartnik, E.; Bonnet-Bidaud, A.; Kowalska, I.; Pieniazek, N.J. New leucine auxotrophs of *Aspergillus nidulans*. *Acta Microbiol. Pol.* **1980**, *29*, 29-33.
7. Wiegel, J. Alpha-isopropylmalate synthase as a marker for the leucine biosynthetic pathway in several Clostridia and in *Bacteroides fragilis*. *Arch. Microbiol.* **1981**, *130*, 385-390.
8. Wiegel, J.; Schlegel, H.G. Alpha-isopropylmalate synthase from *Alcaligenes eutrophus* H 16 I. Purification and general properties. *Arch. Microbiol.* **1977**, *112*, 239-246.
9. Nouwen, N.; Arrighi, J.F.; Cartieaux, F.; Chaintreuil, C.; Gully, D.; Klopp, C.; Giraud, E. The role of rhizobial (NifV) and plant (Fen1) homocitrate synthases in *Aeschynomene*/photosynthetic *Bradyrhizobium* symbiosis. *Sci. Rep.* **2017**, *7*, e448.
10. Koon, N.; Squire, C.J.; Baker, E.N. Crystal structure of leua from *Mycobacterium tuberculosis*, a key enzyme in leucine biosynthesis. *Proc. Natl. Acad. Sci. U. S. A.* **2004**, *101*, 8295-8300.
11. Huisman, F.H.; Hunter, M.F.; Devenish, S.R.; Gerrard, J.A.; Parker, E.J. The C-terminal regulatory domain is required for catalysis by *Neisseria meningitidis* alpha-isopropylmalate synthase. *Biochem. Biophys. Res. Commun.* **2010**, *393*, 168-173.
12. Larson, E.M.; Idnurm, A. Two origins for the gene encoding alpha-isopropylmalate synthase in fungi. *PLoS One* **2010**, *5*, e11605.
13. Que, Y.; Yue, X.; Yang, N.; Xu, Z.; Tang, S.; Wang, C.; Lv, W.; Xu, L.; Talbot, N.J.; Wang, Z. Leucine biosynthesis is required for infection-related morphogenesis and pathogenicity in the rice blast fungus *Magnaporthe oryzae*. *Curr. Genet.* **2020**, *66*, 155-171.
14. Beltzer, J.P.; Morris, S.R.; Kohlhaw, G.B. Yeast Leu4 encodes mitochondrial and nonmitochondrial forms of alpha-isopropylmalate synthase. *J. Biol. Chem.* **1988**, *263*, 368-374.
15. Casalone, E.; Barberio, C.; Cavalieri, D.; Polsinelli, M. Identification by functional analysis of the gene encoding alpha-isopropylmalate synthase II (Leu9) in *Saccharomyces cerevisiae*. *Yeast* **2000**, *16*, 539-545.
16. Steyer, J.T.; Downes, D.J.; Hunter, C.C.; Migeon, P.A.; Todd, R.B. Duplication and functional divergence of branched-chain amino acid biosynthesis genes in *Aspergillus nidulans*. *mBio* **2021**, *12*, e0076821.
17. Diebold, R.; Schuster, J.; Daschner, K.; Binder, S. The branched-chain amino acid transaminase gene family in *Arabidopsis* encodes plastid and mitochondrial proteins. *Plant Physiol.* **2002**, *129*, 540-550.
18. Berger, B.J.; English, S.; Chan, G.; Knodel, M.H. Methionine regeneration and aminotransferases in *Bacillus subtilis, Bacillus cereus*, and *Bacillus anthracis*. *J. Bacteriol.* **2003**, *185*, 2418-2431.
19. Chun, C.D.; Brown, J.C.S.; Madhani, H.D. A major role for capsule-independent phagocytosis-inhibitory mechanisms in mammalian infection by *Cryptococcus neoformans*. *Cell Host Microbe* **2011**, *9*, 243-251.
20. Merchant, S.S.; Prochnik, S.E.; Vallon, O.; Harris, E.H.; Karpowicz, S.J.; Witman, G.B.; Terry, A.; Salamov, A.; Fritz-Laylin, L.K.; Marechal-Drouard, L., *et al.* The *Chlamydomonas* genome reveals the evolution of key animal and plant functions. *Science* **2007**, *318*, 245-250.
21. Somers, J.M.; Amzallag, A.; Middleton, R.B. Genetic fine structure of the leucine operon of *Escherichia coli* k-12. *J. Bacteriol.* **1973**, *113*, 1268-1272.
22. Khoei, M.A.; Karimi, M.; Karamian, R.; Amini, S.; Soorni, A. Identification of the complex interplay between nematode-related lncRNAs and their target genes in *Glycine max* l. *Front Plant Sci* **2021**, *12*, e779597.
23. Yennawar, N.; Dunbar, J.; Conway, M.; Hutson, S.; Farber, G. The structure of human mitochondrial branched-chain aminotransferase. *Acta Crystallogr. D Biol. Crystallogr.* **2001**, *57*, 506-515.

24. Colon, M.; Hernandez, F.; Lopez, K.; Quezada, H.; Gonzalez, J.; Lopez, G.; Aranda, C.; Gonzalez, A. *Saccharomyces cerevisiae* Bat1 and Bat2 aminotransferases have functionally diverged from the ancestral-like *Kluyveromyces lactis* orthologous enzyme. *PLoS One* **2011**, *6*, e16099.
25. Maloney, G.S.; Kochevenko, A.; Tieman, D.M.; Tohge, T.; Krieger, U.; Zamir, D.; Taylor, M.G.; Fernie, A.R.; Klee, H.J. Characterization of the branched-chain amino acid aminotransferase enzyme family in tomato. *Plant Physiol.* **2010**, *153*, 925-936.
